# Maternal Immune Activation by Lipopolysaccharide Reduced Parvalbumin Levels and Increased Perineuronal Nets in the Prefrontal Cortex Without Inducing Schizophrenia-like Behavioral Deficits in PV-Cre Long-Evans Rats

**DOI:** 10.3390/ijms27219649

**Published:** 2026-10-28

**Authors:** Iveta Vojtechova, Klara Tuckova, Viera Kutna, Barbora Jelinkova, Katerina Skalova, Maria Lebedeva, Anastasiia Khodak, Tomas Machacek, Tomas Petrasek

**Affiliations:** 1 National Institute of Mental Health, Topolova 748, 25067 Klecany, Czech Republic; iveta.vojtechova@nudz.cz (I.V.); klara.tuckova@nudz.cz (K.T.); viera.kutna@nudz.cz (V.K.); barbora.jelinkova@nudz.cz (B.J.); katerina.skalova@nudz.cz (K.S.); mariia.lebedeva@nudz.cz (M.L.); anastasiia.khodak@nudz.cz (A.K.); 2 Department of Physiology, Faculty of Science, Charles University, Albertov 6, 12800 Prague 2, Czech Republic; 3 Department of Zoology, Faculty of Science, Charles University, Albertov 6, 12800 Prague 2, Czech Republic; 4 Department of Parasitology, Faculty of Science, Charles University, Albertov 6, 12800 Prague 2, Czech Republic; tomas.machacek@natur.cuni.cz

**Keywords:** maternal immune activation, prenatal infection, chronic bacterial infection, lipopolysaccharide, schizophrenia, autism, parvalbumin, PV-Cre, rat strain

## Abstract

Models of maternal immune activation (MIA) often lead to parvalbumin-positive (PV+) interneuron deficits and behavioral alterations resembling autism or schizophrenia. We aimed to characterize the effects of MIA in PV-Cre Long-Evans rats. Pregnant dams received 8 lipopolysaccharide injections (1 mg/kg), on alternating days, starting with gestational day 7. The offspring behavior was tested from postnatal day 5 to adulthood, with tasks assessing motor development, anxiety, exploration, social behavior and sensorimotor gating. Morphological changes in the brain, density of PV+ interneurons and perineuronal nets in the prefrontal cortex, as well as the quantity of PV protein were measured at selected timepoints. We found that MIA in PV-Cre Long-Evans rats failed to elicit the expected schizophrenia- or autism-like deficits. Decrease in PV protein in the prefrontal cortex was present without corresponding decrement of PV+ interneurons, with a paradoxical increase in perineuronal net counts. PV-Cre Long-Evans rat line reacts to the MIA protocol very differently than Wistar rats in previously published studies and is suboptimal for studies of schizophrenia- or autism-like behavioral deficits. Genetic background seems to be an important factor for the outcome in MIA rodent models.

## 1. Introduction

Neuropsychiatric disorders, such as schizophrenia or autism spectrum disorder (ASD), often have unknown etiology. Maternal infection during pregnancy is a known risk factor, as processes related to infection and activation of the immune system of the mother can harmfully affect brain development of her fetus [1,2,3]. Such an infection can be mimicked in laboratory rodents by timed systemic application of a pathogen, or more often a specific immunogenic molecule, such as the polyinosinic:polycytidylic acid (polyI:C), a synthetic analog of viral RNA, or bacterial lipopolysaccharide (LPS) to pregnant dams [4]. The offspring of rodent mothers who experienced maternal immune activation (MIA) show changes on a brain and behavioral level, resembling neuropsychiatric symptoms diagnosed in human patients [5].

LPS belongs among major immunogenic constituents of the outer membrane of gram-negative bacteria, e.g., *Escherichia coli* or *Salmonella*. It triggers a cascade of immune reactions through the activation of Toll-like receptor 4 (TLR4) which lead to the release of pro-inflammatory cytokines, such as tumor necrosis factor (TNF), interleukin (IL)-6 or IL-1beta [6]. It was shown that increased levels of pro-inflammatory cytokines are measurable in the fetal rat brain even after a single injection of LPS to pregnant dams [7], and they persist to adulthood after chronic prenatal exposure [8].

In our previous study, we demonstrated that chronic activation of the immune system of pregnant rat dams, induced by repeated administration of LPS, caused behavioral alterations, such as social and communication impairments, a prepulse inhibition deficit, increased startle reflex and anxiety, in the offspring [9]. In addition, disruption caused by LPS-induced MIA was apparent already in juvenile age of the offspring, as both sexes showed enlargement of the brain and decreased numbers of parvalbumin (PV)-positive interneurons in the frontal cortex [9].

The correct amount of interneuronal PV or number of PV-positive interneurons in the brain is essential for the proper functioning of neuronal networks and its alteration could result in a fatal disruption of excitation/inhibition balance and in discoordination [10]. Therefore, a malfunction of PV-positive interneurons might be considered one of the significant mechanisms behind the subsequent manifestation of behavioral disruptions in rodent models [11,12,13], and possibly the cause of at least some symptoms of mental disorders in humans [14,15,16].

**Figure 1:**
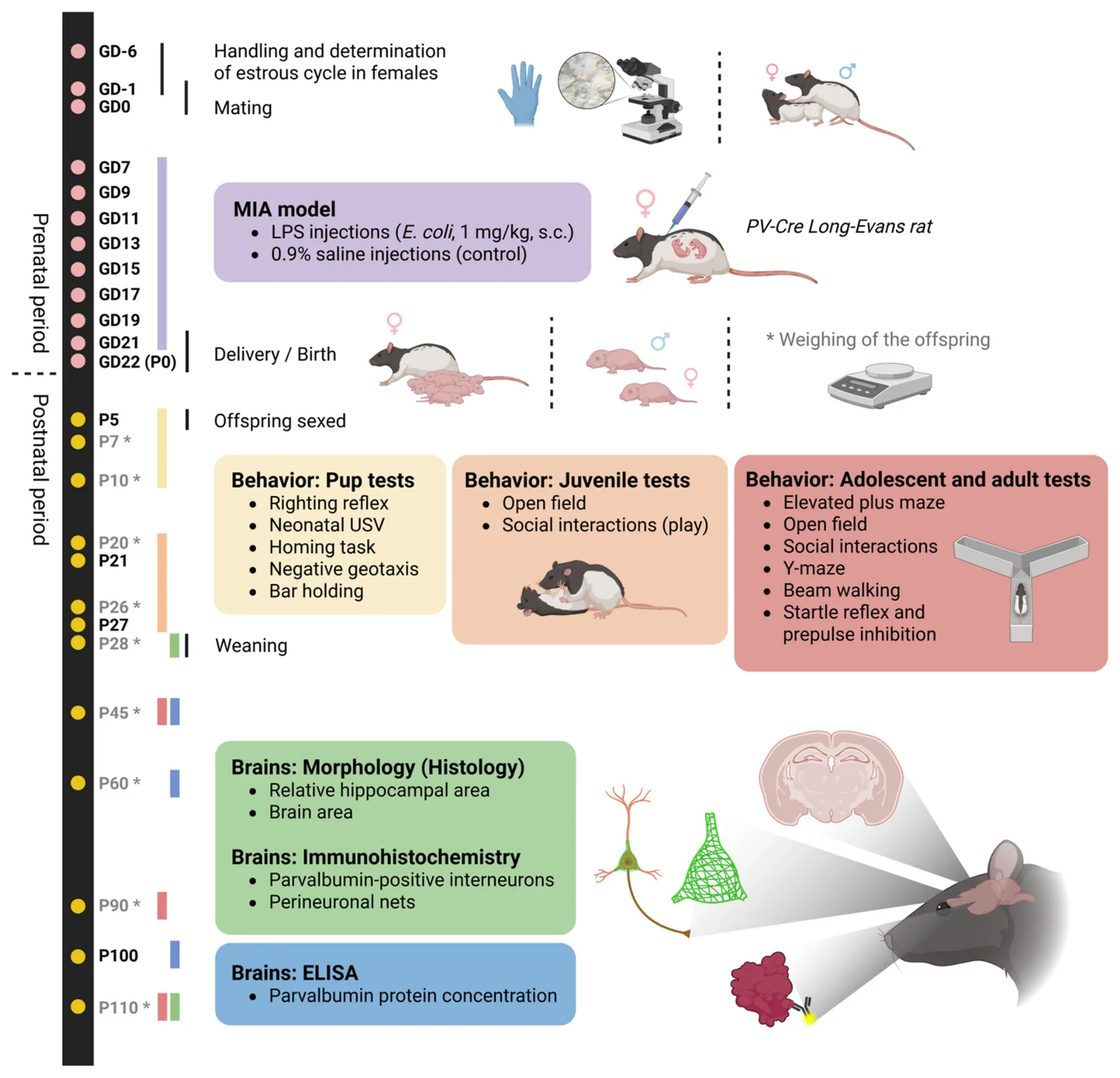
Timeline of manipulations and individual experiments. After several days of handling and determination of the estrous cycle, females and males of PV-Cre rats were mated for 24 h (gestational day GD0). During the gestation period, pregnant females received 8 subcutaneous (s.c.) injections of bacterial lipopolysaccharide (LPS; *E. coli*, L-3755, Serotype 026:B6, 1 mg/kg) to induce maternal immune activation (MIA), or 0.9% saline solution (control), administered GD7-GD21 (every other day). Offspring of the treated dams underwent a comprehensive battery of behavioral tests at various developmental stages: pups (postnatal day P5, P7, P10), juveniles (P20, P21, P26, P27), adolescents (P45) and adults (P90, P110). Brains of the offspring were analyzed for morphology (P28, P110), parvalbumin interneurons and perineuronal nets (P28, P110) and parvalbumin protein concentrations (P45, P60, P100). Grey asterisks indicate when the offspring was weighted. Created in BioRender. Vojtechova, I. (2026) https://BioRender.com/5rehf55 (accessed on 24 August 2026).

The above-mentioned hypothesis led us to establish the MIA model in transgenic Parvalbumin-Cre (PV-Cre) knock-in rats, with the vision of enabling future targeted chemogenetic modulation of PV-positive interneuron activity in MIA and control animals. The model of chronic prenatal exposure to LPS, which we describe in this study, has been well established in albinotic Wistar rats [9,17,18]. However, manifestation of such intervention could be influenced by a huge number of factors, including genetic background [19,20]. Therefore, we characterized impacts of chronic LPS-induced MIA using the pigmented PV-Cre rats with the Long-Evans strain background. In the present study, Cre recombinase was not employed; therefore, the animals should act as standard Long-Evans. The animals underwent a comprehensive battery of behavioral tests, from pup age to adulthood (Figure 1), with the aim to differentiate ASD-like and schizophrenia-like deficits by specific developmental patterns. In addition, parvalbumin expression in their brains, density of PV-positive interneurons and occurrence of perineuronal nets (PNN) were quantified at several age points.

## 2. Results

### 2.1. LPS Triggered a Systemic Immune Reaction

**Figure A1:**
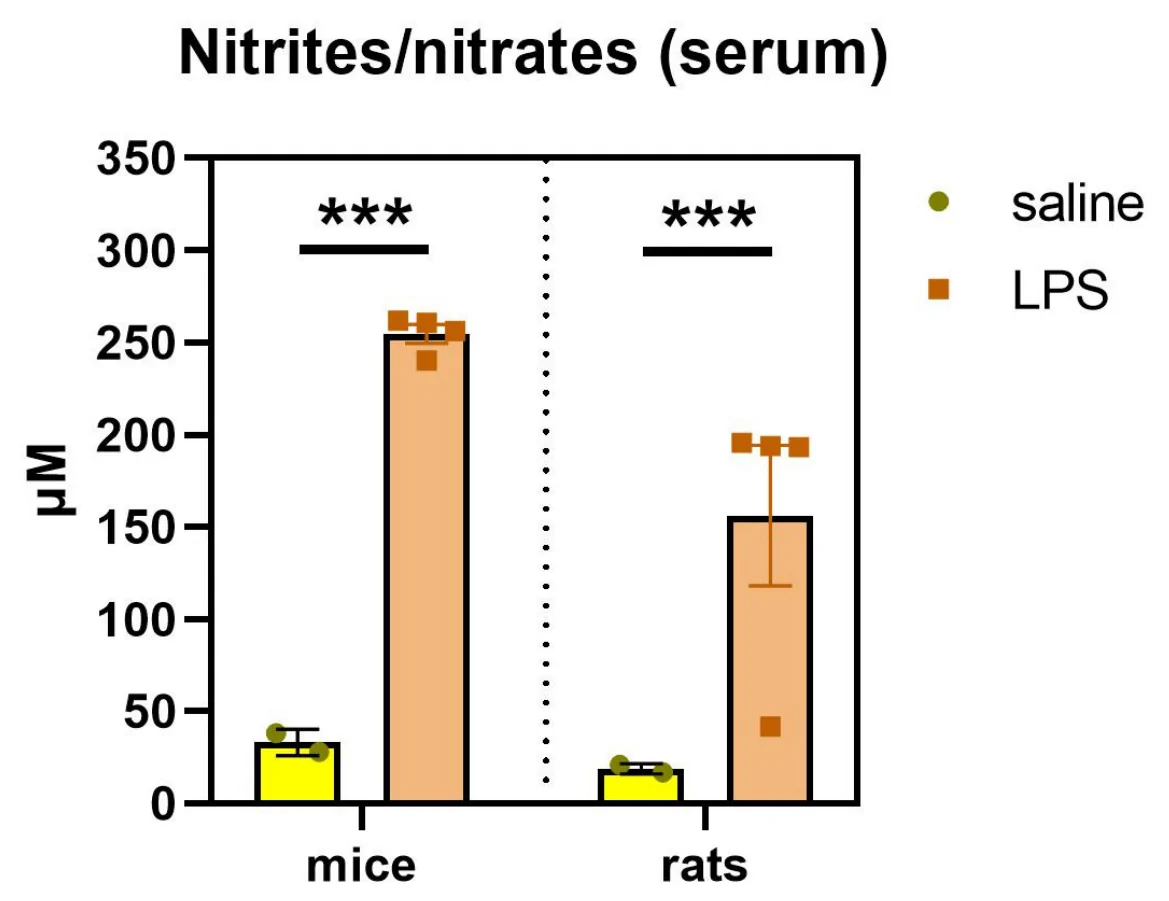
Lipopolysaccharide (LPS) triggered an immune reaction. Serum nitrites/nitrate levels were significantly elevated by LPS injection in both mice and rats. The bars show mean ± SEM; the dots show individual values. Number of animals per group: *n* (mice saline) = 2; *n* (mice LPS) = 4; *n* (rats saline) = 2; *n* (rats LPS) = 4. *** *p* < 0.001.

We verified that LPS used in our study is able to trigger a systemic inflammatory immune reaction. Administration of LPS caused the significant elevation of serum nitrites/nitrate levels in animals (F(1, 9) = 34.27; *p* < 0.001) (Figure A1). Holm–Šidák’s multiple-comparison test confirmed the significance in both mice (*p* < 0.001) and rats (*p* < 0.001).

### 2.2. Prenatal LPS Caused Mild Effects on Neonatal Vocalization and Sensorimotor Development with No Impact on Weight

**Figure A2:**
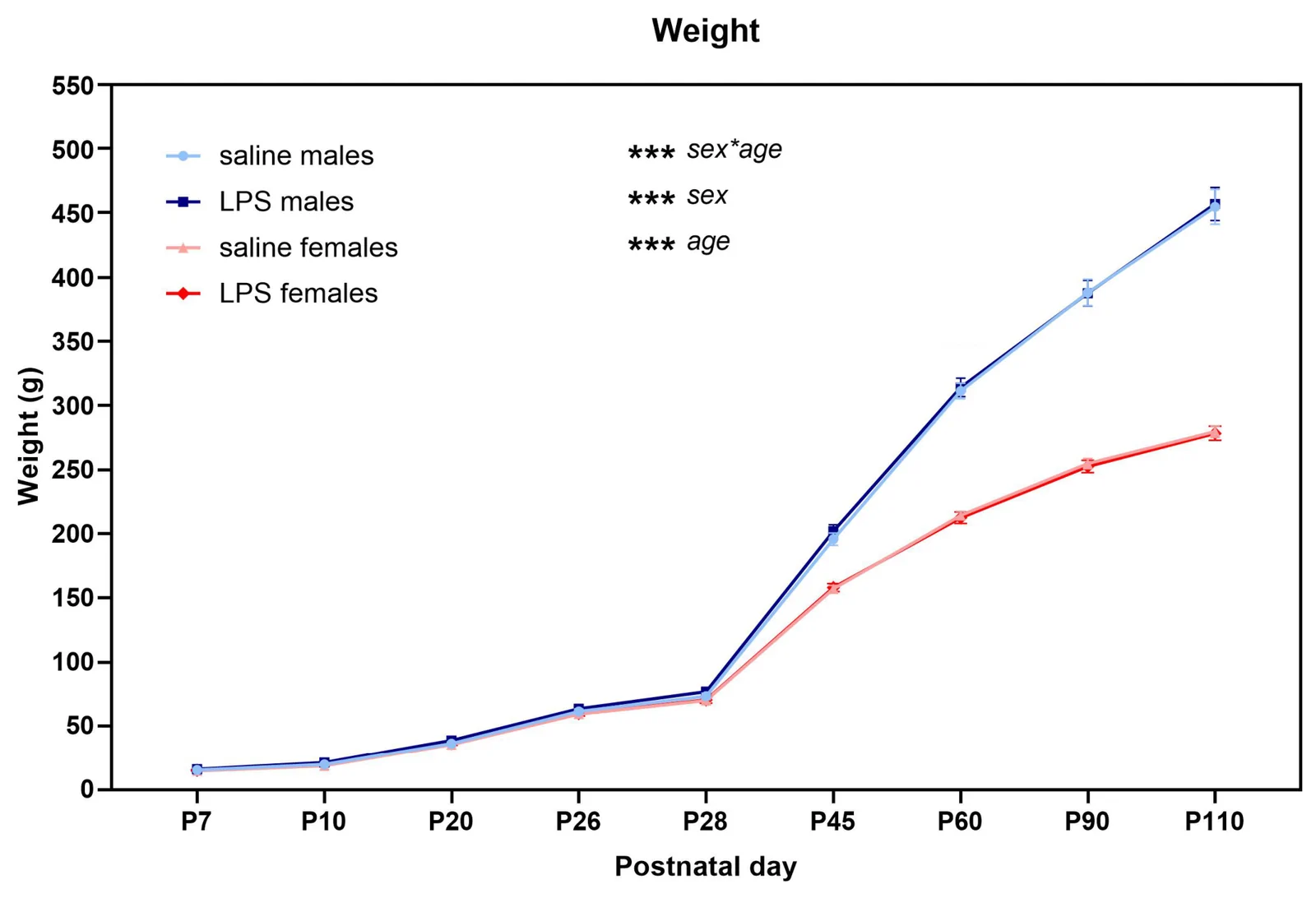
The weight of the offspring. Prenatal lipopolysaccharide (LPS) had no effect on the weight of the offspring. The weight was higher and increased faster in males compared to females already from pup age to adulthood. The dots show mean ± SEM. Number of animals per group: *n* = 18. *** *p* < 0.001.

The offspring was weighed every day during the behavioral testing from pup age to adulthood, always after performing the test. The weight of animals that originated from mothers who received LPS injections did not differ from controls. Linear mixed models (LMM) analysis showed the significant effect of age (F(8, 544) = 22,172.7; *p* < 0.001), sex (F(1, 55.3) = 257.934; *p* < 0.001) and the interaction sex×age (F(8, 544) = 128.183; *p* < 0.001) (Figure A2).

**Figure 2:**
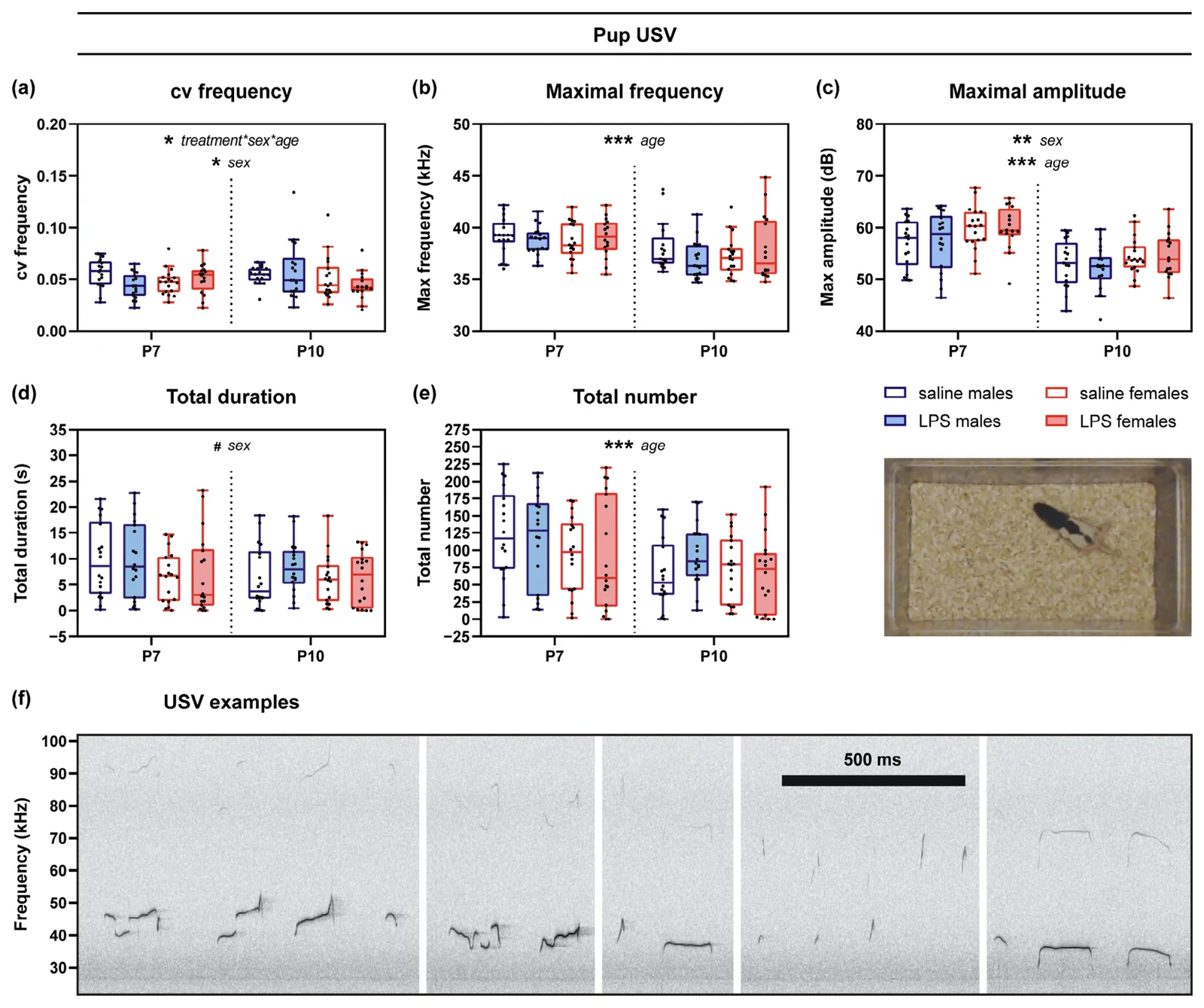
Neonatal ultrasonic vocalization (USV) at postnatal days P7 and P10. (**a**) For the coefficient of variation of peak frequencies (cv frequency), there was a significant main effect of sex, and significant triple interaction treatment×sex×age showing the opposite effect of prenatal LPS treatment on males and females, which was also dependent on age. Specifically, cv frequency was decreased in LPS males and increased in LPS females in comparison with controls at P7. (**b**) Maximal frequency decreased from P7 to P10. (**c**) The maximal amplitude was not affected by LPS, but the analysis revealed that females produced louder vocalizations than males, and younger animals were louder than the older ones. (**d**) LPS did not affect the total duration of USV. (**e**) No effect of LPS was found in the total number of USV, but it decreased with age. (**f**) Selected spectrograms showing the variety of neonatal USV. Boxplots show the median (horizontal line), first and third quartiles (lower and upper box boundaries), and whiskers extending to the minimum and maximum values within 1.5× the interquartile range. Dots represent individual data points. Number of animals per group: *n* = 18. *** *p* < 0.001; ** *p* < 0.01; * *p* < 0.05; # *p* = 0.05–0.10.

We measured the ultrasonic vocalization (USV) of neonates isolated from their mothers at postnatal day (P) 7 and P10. The main effect of LPS was not apparent in any of the measured parameters. For the cv frequency (coefficient of variation of peak frequencies), the analysis revealed significant effect of sex (F(1, 56) = 4.792; *p* = 0.033) and significant interaction treatment×sex×age (F(1, 66.3) = 4.561; *p* = 0.036), showing that the complexity of frequency modulation was oppositely affected by LPS in males and females at P7, but not at P10 (Figure 2a). For the maximal frequency (call frequency at the moment of highest amplitude), the analysis showed the significant effect of age (F(1, 65.6) = 41.751; *p* < 0.001), frequency was lower in older pups compared to younger pups (Figure 2b). Further, the analysis revealed a significant effect of sex (F(1, 55.2) = 10.325; *p* = 0.002) and age (F(1, 65.8) = 80.645; *p* < 0.001) in the maximal amplitude with louder vocalizations in females compared to males and in younger compared to older pups (Figure 2c). Total number of USV was significantly lower in older pups (effect of age, F(1, 68) = 11.798; *p* = 0.001) (Figure 2e). Moreover, a trend towards shorter total duration of USV was revealed in females compared to males (non-significant effect of sex, F(1, 56.1) = 3.257; *p* = 0.076) (Figure 2d).

**Figure 3:**
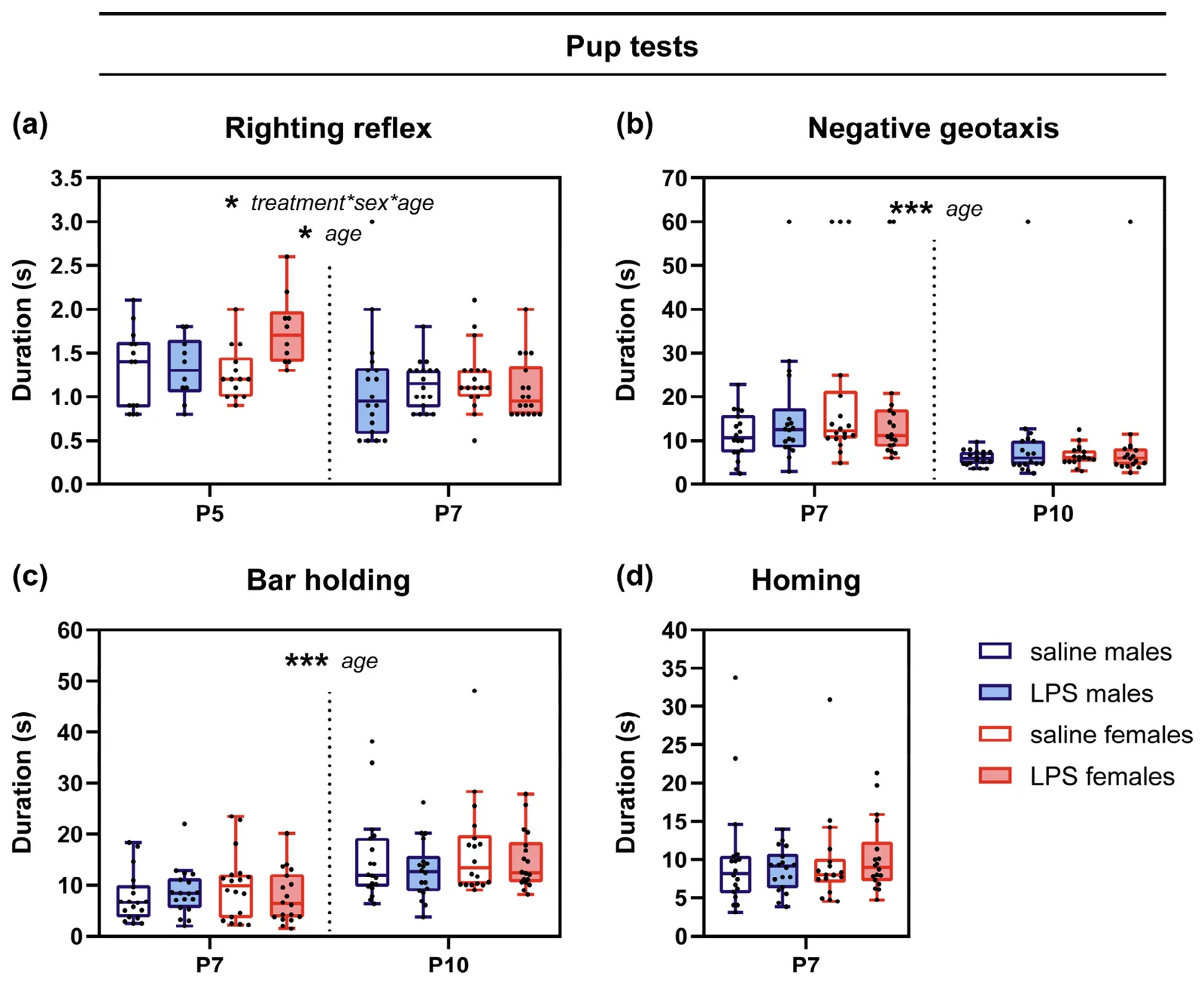
Tests of sensorimotor development at postnatal days P5, P7 and P10. No significant effect of prenatal LPS exposure was found in righting reflex (**a**), negative geotaxis (**b**), bar-holding test (**c**), and homing task (**d**). Nevertheless, righting response (**a**) was delayed in P5 LPS females (significant interaction treatment×sex×age). Older pups manifested faster righting reflex (**a**), faster rotation (**b**), better grip strength and sensorimotor coordination (**c**) compared with younger pups. Boxplots show the median (horizontal line), first and third quartiles (lower and upper box boundaries), and whiskers extending to the minimum and maximum values within 1.5× the interquartile range. Dots represent individual data points. Number of animals per group: *n* = 18. *** *p* < 0.001; * *p* < 0.05.

In addition, rat pups underwent a set of tests measuring their sensorimotor development. In the test of righting reflex, the significant effect of age (F(1, 51) = 6.743; *p* = 0.012), significant interaction treatment×sex×age (F(1, 62.1) = 6.896; *p* = 0.011) and post hoc tests showed that younger pups needed longer time to turn over to the prone position than the older ones, with the 5-day-old LPS females being the slowest (Figure 3a). In the negative geotaxis and bar-holding tests, prenatal LPS exposure did not affect sensorimotor coordination, or pup’s grip strength, respectively (Figure 3b,c). However, the analysis revealed a significant effects of age (negative geotaxis: F(1, 68) = 70.493; *p* < 0.001; bar holding: F(1, 68) = 52.672; *p* < 0.001) as older animals were able to rotate faster or hang on a horizontal rod for a longer time, respectively (Figure 3b,c). The ability to find the home nest in the homing task was not affected by any of the measured factors (Figure 3d).

In conclusion, prenatal LPS exposure had no effect on weight of the offspring and caused only mild changes in communication of pups and their sensorimotor development.

### 2.3. Prenatal LPS Altered Social Play and Self-Grooming Behavior but Not Locomotor Activity and Anxiety in Juvenile Rats

**Figure 4:**
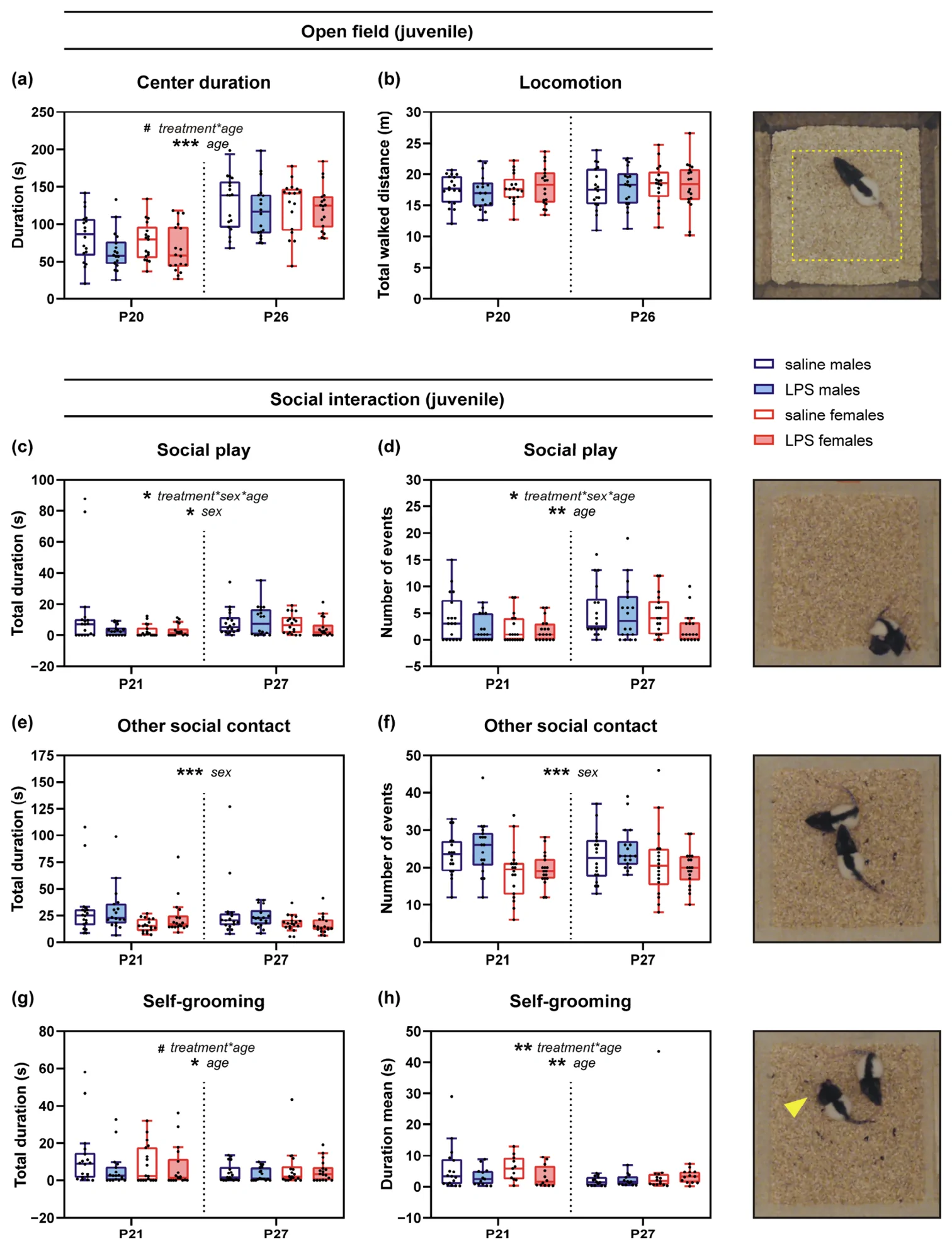
Open field (OF) test and social interaction at postnatal days P20, P21, P26 and P27. (**a**) Older juveniles spent more time in the center of the apparatus (yellow dotted box in the photo represents the center zone). There was also a trend toward treatment×age interaction. (**b**) Locomotor activity in the OF was not altered by prenatal LPS exposure. (**c**) The offspring of LPS-treated dams spent less time playing than the control offspring but the effect was dependent on sex and age (significant interaction treatment×sex×age). In addition, the time spent playing was longer in males than females. (**d**) The number of social play events was decreased by LPS depending on sex and age (significant interaction treatment×sex×age). Moreover, the number of social play occurrences increased with age. (**e**,**f**) Total duration and the number of other social contact were not changed by prenatal LPS treatment. However, males spent more time in social exploration and made a higher number of social contacts compared to females. (**g**) Mildly shorter total duration of self-grooming behavior in LPS-treated rats at P21 was suggested by a trend in interaction treatment×age. Grooming duration significantly decreased with age. (**h**) Shorter self-grooming events in LPS-exposed rats at P21 but not P27 were revealed by significant interaction treatment×age, grooming bouts also grew shorter with age. Yellow arrowhead in the photo aims at the rat performing self-grooming. Box plots show the median (horizontal line), first and third quartiles (lower and upper box boundaries), and whiskers extending to the minimum and maximum values within 1.5× the interquartile range. Dots represent individual data points. Number of animals per group: *n* = 18 (one OF recording from the female saline group was lost due to technical error). *** *p* < 0.001; ** *p* < 0.01; * *p* < 0.05; # *p* = 0.05–0.10.

Juvenile rats were tested in an open field (OF) test to measure anxiety and locomotor activity. For center duration, we found a weak trend in interaction treatment×age (F(1, 67) = 2.906; *p* = 0.093) showing that the offspring of LPS-treated mothers spent mildly less time in the center than control offspring at P20 but not P26 (Figure 4a). Moreover, older juveniles spent significantly more time in the center than the younger ones (F(1, 67) = 144.995; *p* < 0.001) (Figure 4a). The total walked distance in OF was comparable (Figure 4b).

In juveniles, we also explored social behavior. Rats prenatally exposed to LPS were engaged in social play less often and for a shorter time compared to controls, but the difference was dependent on sex and age, as it was demonstrated by significant interactions treatment×sex×age (number of events: F(1, 68) = 5.010; *p* = 0.028; total duration: F(1, 68) = 5.566; *p* = 0.021) (Figure 4c,d). However, we should note that only one pair of control males engaged in intensive play with a social partner while other animals were much less playful. Statistical analysis also showed a significant effect of sex (F(1, 24.7) = 4.574; *p* = 0.043) and age (F(1, 68) = 8.321; *p* = 0.005), showing that males played for a longer time than females (Figure 4c) and that older animals were willing to play more often than the younger ones (Figure 4d). Higher frequency of playful behavior in rat males is rather typical [21]. Other social contact excluding social play was not affected by LPS exposure and the only significant effect of sex revealed that females spent less time in social exploration than males (F(1, 23.9) = 17.296; *p* < 0.001) (Figure 4e) and made fewer social contacts (F(1, 24.7) = 16.578; *p* < 0.001) (Figure 4f). Concerning the non-social behavior measured during the social play test, prenatal LPS exposure caused shorter bouts of self-grooming behavior in P21-old juveniles which was shown by significant interaction treatment×age (F(1, 52.9) = 7.967; *p* = 0.007) and the post hoc test (Figure 4h), and supported by a trend to shorter total duration of self-grooming (treatment×age: F(1, 68) = 2.985; *p* = 0.089) (Figure 4g). In addition, older juveniles did shorter bouts of self-grooming (F(1, 52.8) = 7.686; *p* = 0.008) and for a shorter total time (F(1, 68) = 4.000; *p* = 0.049) (Figure 4g,h). LPS had no significant effect on digging in the bedding.

In conclusion, LPS-exposed juveniles showed changes in social play and self-grooming behavior, but the specificity of changes was dependent on sex and age. Locomotor activity and anxiety were not substantially affected by LPS.

### 2.4. Prenatal LPS Changed Risk Assessment Behavior, Locomotor Activity and Social Behavior in Adolescents and Adults Depending on Sex and Age

Offspring rats in the age of adolescence and adulthood underwent a series of behavioral tests measuring various aspects of behavior, including anxiety, exploration, locomotor activity, social behavior, memory, motor coordination and sensorimotor gating.

**Figure 5:**
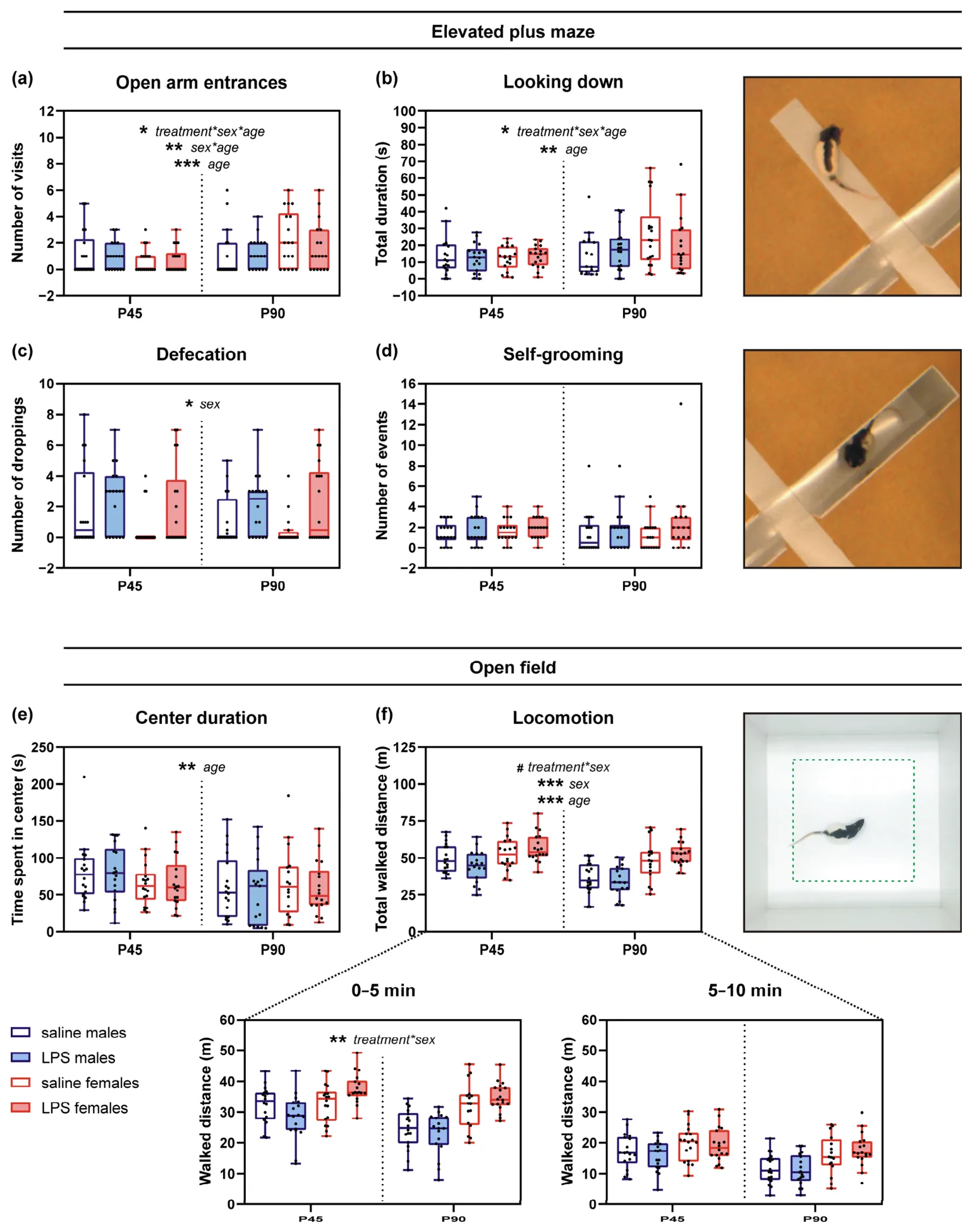
Elevated plus maze (EPM) and open field (OF) at postnatal days P45 and P90. (**a**) For open-arm entrances, triple statistical interaction treatment×sex×age showed that the effect of prenatal LPS treatment was significant, but the direction of the effect was dependent on sex and age. In addition, males entered the open arms more often than females at P45, but females did so more often than males at P90. Moreover, adult offspring visited the open arms more often than adolescents. Unfortunately, the number of entrances into the open arms was very low in general. (**b**) At P90, the duration of looking-down behavior was longer in LPS-treated males while shorter in LPS-treated females in comparison to controls, but not at P45 (significant interaction treatment×sex×age). Additionally, adult animals looked down from the maze for a longer time than adolescents. (**c**) Defecation was significantly higher in males compared to females, but it was not significantly changed in LPS-exposed rats compared to control rats. (**d**) Prenatal LPS exposure did not affect the frequency of self-grooming behavior. (**e**) LPS animals spent a comparable time in the center of the OF (represented by the green dotted box in the photo) as control animals, and the time in the center decreased with age. (**f**) Prenatal LPS treatment significantly affected walked distance in the arena and the effect differed in males and females (trend in the interaction treatment×sex for the 10-min session, with the same interaction being significant for the first 5 min of the session). The walked distance was shorter in LPS males but longer in LPS females compared to control rats. Furthermore, females and younger rats exhibited higher locomotor activity than males and older rats, respectively. Box plots show the median (horizontal line), first and third quartiles (lower and upper box boundaries), and whiskers extending to the minimum and maximum values within 1.5× the interquartile range. Dots represent individual data points. Number of animals per group: *n* = 18. *** *p* < 0.001; ** *p* < 0.01; * *p* < 0.05; # *p* = 0.05–0.10.

In the elevated plus maze (EPM), statistical analysis of the frequency of open arm entrances revealed significant interaction treatment×sex×age (F(1, 68) = 4.715; *p* = 0.033), but post hoc tests did not reveal any specific significant difference (Figure 5a). However, we should note that the total number of open arm entrances was very low in all the groups and a high number of animals did not enter the open arms at all. The generally high levels of anxiety-like behavior have introduced a strong floor effect constraining the variability of this measure, and the result should therefore be interpreted with caution. Moreover, there was a significant interaction sex×age (F(1, 68) = 10.565; *p* = 0.002) and significant effect of age (F(1, 68) = 11.834; *p* = 0.001), showing that open arms were more often visited by males at P45, but by females at P90, and that frequency of open arm visits increased in older animals (Figure 5a). Concerning risk assessment behavior, significant interaction treatment×sex×age (F(1, 68) = 5.888; *p* = 0.018) showed that prenatal LPS had a different impact on duration of looking down from the maze in males and females at P45 and P90 (Figure 5b). Specifically, LPS-exposed males looked down from the maze for a longer time compared to control males, while LPS-exposed females looked down for a shorter time compared to control females at P90. In addition, the significant effect of age (F(1, 68) = 9.085; *p* = 0.004) indicated increased duration of looking-down behavior in older rats compared to younger rats (Figure 5b). This behavioral category is similar but not synonymous with the traditional “head dipping”, as the maze was only viewed from above, and actual lowering of the head below the level of the maze floor could not be reliably determined. We emphasize that this behavior is not a measure of anxiety, but rather risk assessment or approach/avoidance conflict measure [22]. None of the other measured parameters showed a significant effect of LPS treatment.

LPS did not affect anxiety measured by time spent in the central part of the OF; only adults spent less time in the center compared to adolescents (effect of age: F(1, 66.8) = 7.524; *p* = 0.008) (Figure 5e). However, LPS-exposed rats differed from saline-treated rats in locomotor activity and the difference was dependent on sex. For total walked distance during a whole session, the interaction treatment×sex remained an insignificant trend (F(1, 57.1) = 3.328; *p* = 0.073) (Figure 5f), but was significant in the first half of the session (F(1, 57) = 7.178; *p* = 0.010), which implies that LPS males walked a shorter distance, while LPS females walked a longer distance, in comparison to controls, during the initial exploration of the OF. The significant effect of sex (F(1, 57.1) = 33.606; *p* < 0.001) and age (F(1, 66.3) = 47.384; *p* < 0.001) revealed lower activity in males compared to females, and in adults compared to adolescents (Figure 5f).

**Figure 6:**
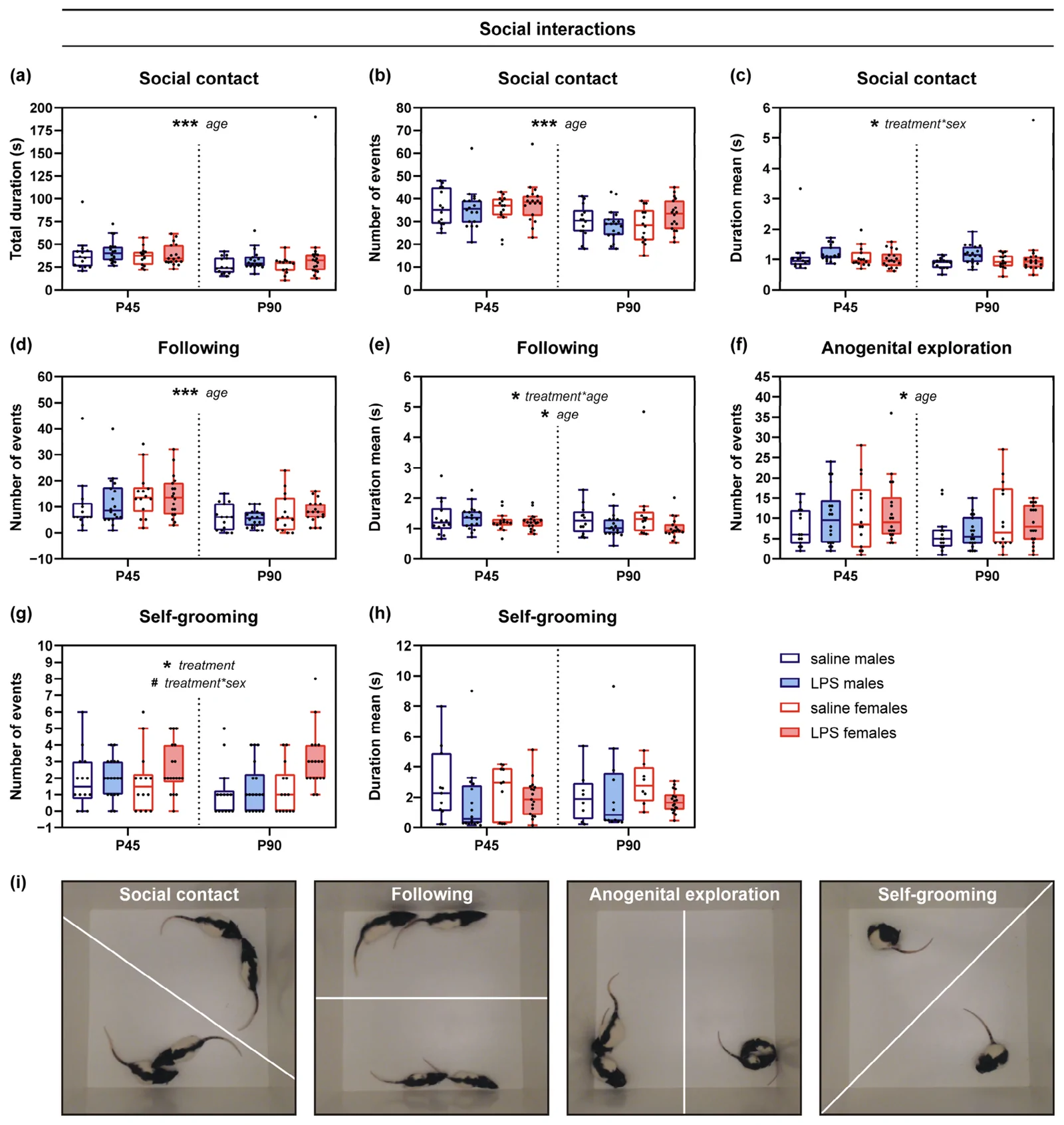
Social interaction (SI) at postnatal days P45 and P90. (**a**,**b**) Total duration and frequency of social contacts was longer in adolescents than adults. (**c**) Prenatal LPS treatment led to increase in average duration of a non-anogenital social contact, especially in males (significant interaction treatment×sex). (**d**) LPS did not affect the frequency of following the social partner. Nevertheless, younger rats followed the social partner more often than older rats. (**e**) Prenatal exposure to LPS caused the shorter average duration of one following occurrence at P90 (significant interaction treatment×age). Following episodes were longer in adolescent than adult animals (effect of age). (**f**) Anogenital exploration was not changed by LPS. However, the frequency of anogenital exploration was higher in adolescents compared to adults. (**g**) Offspring of LPS-treated dams showed higher frequency of self-grooming behavior than offspring of control dams. (**h**) The average duration of one self-grooming occurrence was not significantly affected by any of the studied factors. (**i**) Screenshots illustrating specific behaviors. Box plots show the median (horizontal line), first and third quartiles (lower and upper box boundaries), and whiskers extending to the minimum and maximum values within 1.5× the interquartile range. Dots represent individual data points. Number of animals per group: *n* (males saline) = 14; *n* (males LPS) = 18; *n* (females saline) = 14; *n* (females LPS) = 18. *** *p* < 0.001; * *p* < 0.05; # *p* = 0.05–0.10.

Social behavior was measured in direct social interaction (SI) with an unknown partner from the same group. Non-anogenital social contacts were affected by prenatal LPS exposure depending on sex. The male offspring of LPS-treated dams showed significantly longer average duration of a social contact compared to controls (significant interaction treatment×sex: F(1, 25.9) = 5.439; *p* = 0.028) (Figure 6c). In addition, younger animals contacted social partners more often (F(1, 60) = 28.667; *p* < 0.001) and for a longer time (F(1, 60) = 24.292; *p* < 0.001) than the older ones (Figure 6a,b). Concerning the following of the social partner, the average duration of one following event was significantly shorter in LPS animals, but only in adulthood (treatment×age: F(1, 59.3) = 6.155; *p* = 0.016) (Figure 6e). Moreover, the number of following occurrences (F(1, 60) = 42.658; *p* < 0.001) and average duration of one following occurrence (F(1, 59.3) = 5.437; *p* = 0.023) were higher in adolescents compared to adults (Figure 6d,e). However, LPS treatment had no effect on anogenital exploration. The only significant effect of age revealed that younger rats explored the anogenital area of their social partners more often (F(1, 60) = 6.348; *p* = 0.014) in comparison to older rats (Figure 6f). Interestingly, LPS-exposed offspring self-groomed more often than saline-exposed offspring (F(1, 11.1) = 5.577; *p* = 0.038) (Figure 6g). Preliminary analysis of ultrasonic vocalizations during SI sessions revealed significant effects of treatment, sex and age, with LPS rats vocalizing more than controls, males vocalizing more than females, and adult rats vocalizing more than adolescents. The same pattern was present in both total number of calls and the total duration of ultrasonic vocalization. Detailed analysis of vocalization data will be presented in a follow-up paper.

**Figure 7:**
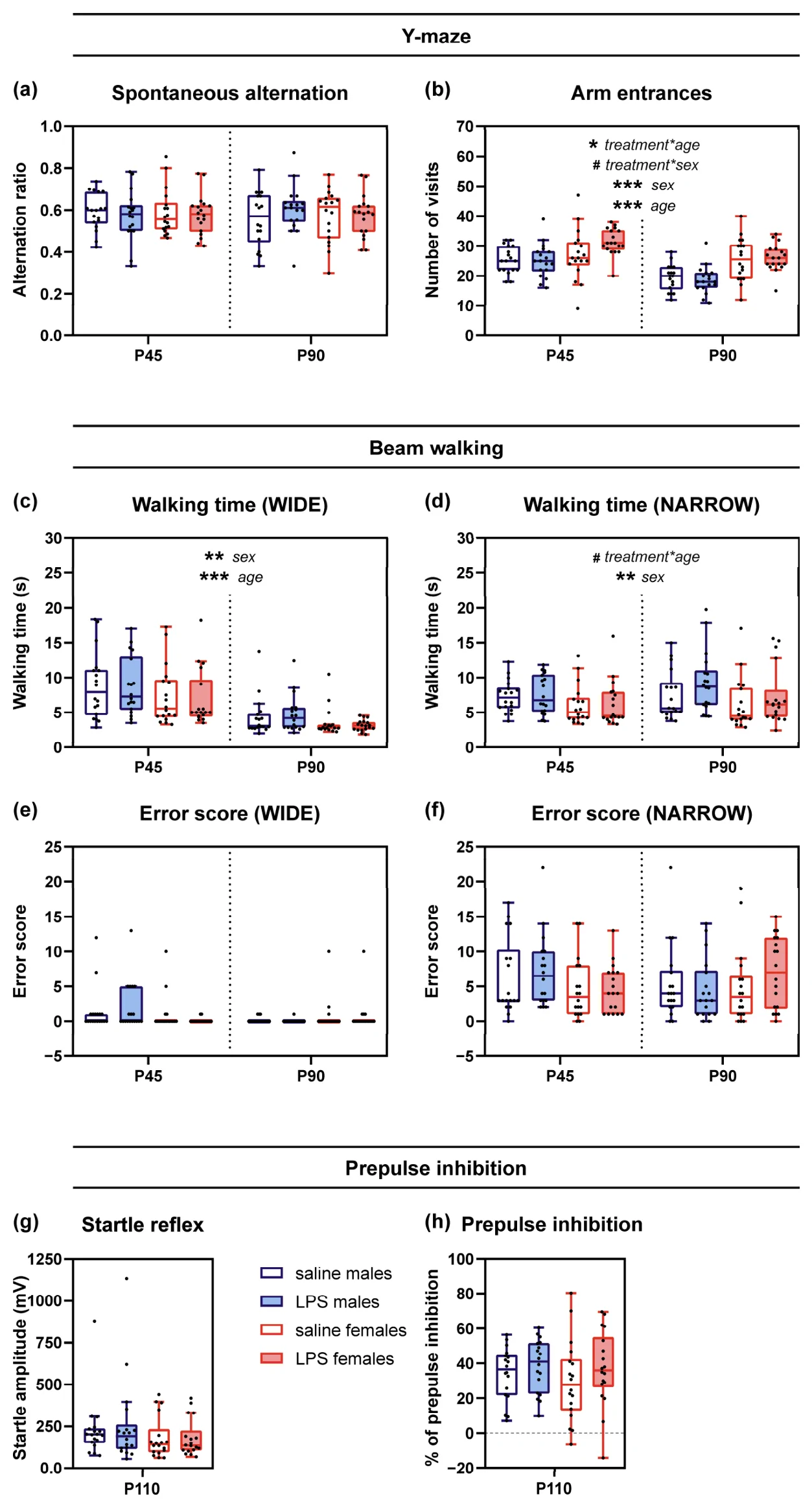
Y-maze task, beam walking (BW), startle reflex and prepulse inhibition (PPI) at postnatal days P45, P90 and P110. (**a**) Prenatal LPS treatment had no effect on spontaneous alternation in the Y-maze. (**b**) LPS-exposed rats, especially females at P45, visited the arms of the Y-maze more often than controls (interaction treatment×age). In addition, arm visits were decreased in males and in older animals. (**c**,**d**) LPS-treated animals showed no difference in the time needed to traverse the wide beam, but they showed a tendency to be slower on the narrow beam at P90, in comparison to saline-treated rats (trend-level interaction treatment×age). In addition, females traversed the wide and narrow beam faster than males. Similarly, older rats traversed the wide beam faster than younger rats. (**e**,**f**) LPS had no significant effect on the error score. Prenatal experience with LPS had no significant effect on the startle reflex (**g**) and prepulse inhibition (**h**). The horizontal dashed line divides prepulse inhibition (above the line) and prepulse potentiation (below the line). Box plots show the median (horizontal line), first and third quartiles (lower and upper box boundaries), and whiskers extending to the minimum and maximum values within 1.5× the interquartile range. Dots represent individual data points. Number of animals per group: *n* = 18. *** *p* < 0.001; ** *p* < 0.01; * *p* < 0.05; # *p* = 0.05–0.10.

In the Y-maze task, working memory measured as spontaneous alternation was not significantly impaired by prenatal LPS experience (Figure 7a). However, statistical analysis found significant interaction treatment×age (F(1, 68) = 4.139; *p* = 0.046) and a trend in interaction treatment×sex (F(1, 58.8) = 3.068; *p* = 0.085) in the total number of arm visits and we could suggest that LPS-exposed animals (especially females at P45, Figure 7b) were more active compared to the others. The significant effect of sex (F(1, 60) = 25.948; *p* < 0.001) showed lower activity in males compared to females, and the number of visits was also decreased in older animals (F(1, 68) = 60.637; *p* < 0.001) (Figure 7b).

We investigated the impact of prenatal exposure to LPS on motor coordination of the offspring in the beam-walking test. Although there was no effect of LPS on the time needed to traverse the wide beam, we found a trend towards longer time needed to traverse the narrow beam in LPS-exposed rats at P90 (interaction treatment×age: F(1, 68) = 3.345; *p* = 0.072) (Figure 7c,d). The effect of sex in the walking time was found in the case of the wide beam (F(1, 60.1) = 9.414; *p* = 0.003) as well as the narrow beam (F(1, 59.5) = 9.450; *p* = 0.003) and showed that females walked faster than males in both cases (Figure 7c,d). In the case of the wide beam, adult animals walked faster than adolescents (F(1, 67.7) = 82.965; *p* < 0.001) (Figure 7c). However, no significant effect of prenatal LPS was revealed in the error score (Figure 7e,f) suggesting there was no serious impairment of motor coordination.

Sensorimotor gating was measured by a prepulse inhibition task in the adult offspring. Neither startle reaction to loud acoustic stimulus (Figure 7g), nor prepulse inhibition (PPI) was affected by prenatal LPS treatment (Figure 7h).

In conclusion, prenatal treatment by LPS caused behavioral changes in adolescent and adult offspring. Specifically, LPS altered risk assessment behavior, grooming and locomotor activity, as well as social and communicative behaviors. However, the direction of these changes was dependent on sex and age. On the other hand, LPS had no effect on anxiety, startle reflex and PPI and did not impair memory and motor coordination.

### 2.5. Relative Hippocampal Area Is Dependent on Sex and Age, but Not LPS Treatment

**Figure 8:**
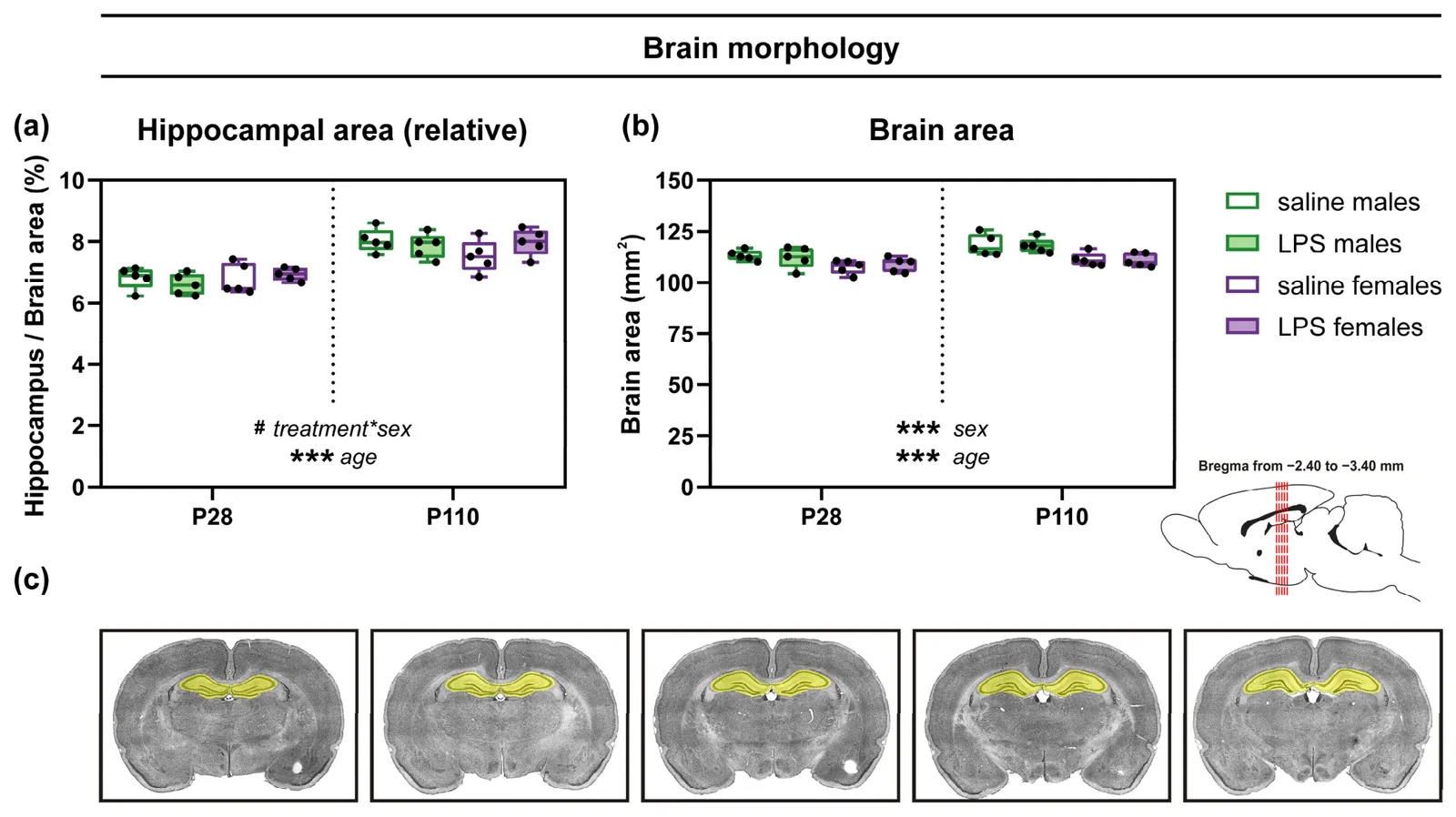
Relative hippocampal area and total brain area at postnatal days P28 and P110. (**a**) Relative hippocampal area tended to be oppositely altered in males and females by prenatal LPS treatment (trend-level interaction treatment×sex). In LPS males, relative hippocampal area size was smaller, whereas in LPS females, it was larger, compared to control animals. Moreover, juveniles had smaller relative hippocampal area size than adults. (**b**) The whole brain area size was not changed by prenatal LPS treatment. Nevertheless, females and juveniles had smaller brain area size than males and adults, respectively. (**c**) Representative tile-scan images showing five consecutive toluidine blue–stained coronal sections from a P28 animal. Box plots show the median (horizontal line), first and third quartiles (lower and upper box boundaries), and whiskers extending to the minimum and maximum values within 1.5× the interquartile range. Dots represent individual data points. Number of animals per group: *n* = 5. *** *p* < 0.001; # *p* = 0.05–0.10.

Apart from the behavioral changes, we explored the effect of prenatal LPS treatment on the size of hippocampal and brain area using classical histology methods. Statistical analysis revealed a trend-level interaction treatment×sex (F(1, 24.9) = 3.701; *p* = 0.066) suggesting opposite effect of LPS on relative hippocampal area for males and females at both ages, P28 and P110 (Figure 8a). Specifically, LPS-treated males had slightly smaller relative hippocampal area size compared to control males, whereas LPS-treated females tended to have larger relative hippocampal area size than control females. The size of the whole brain area was not affected by LPS, but the significant effect of sex (F(1, 22.1) = 33.21; *p* < 0.001) showed smaller brain area size in females compared to males at both ages (Figure 8b). In addition, adult animals had larger total brain area size (F(1, 30.1) = 13.41; *p* < 0.001) (Figure 8b), as well as larger relative hippocampal area size (F(1, 32) = 64.419; *p* < 0.001) (Figure 8a), in comparison to juveniles.

### 2.6. Prenatal LPS Increased Perineuronal Nets but Did Not Disrupt Their Association with PV-Positive Interneurons

**Figure 9:**
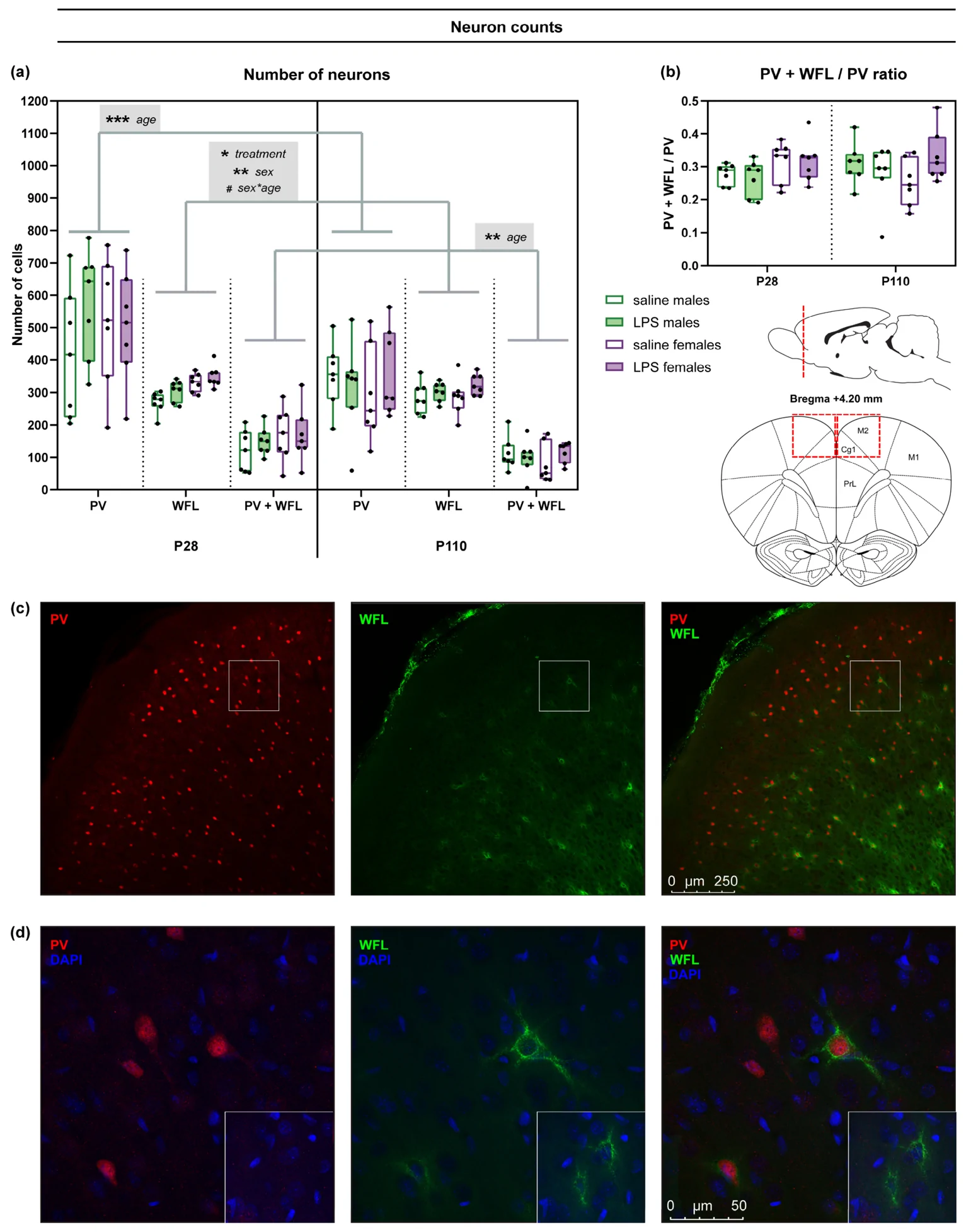
Parvalbumin (PV)-positive interneurons and perineuronal nets stained by Wisteria floribunda lectin (WFL) in the prefrontal cortex at postnatal days P28 and P110. (**a**) The numbers of PV-positive interneurons and PV-positive interneurons enwrapped by perineuronal nets (PV + WFL) were not affected by prenatal LPS exposure, but both were decreased at P110 compared to P28. However, LPS caused an increase in the number of WFL-positive cells. In addition, the number of WFL-positive cells was higher in females, especially at P28. (**b**) Ratio of PV- and WFL-positive versus all PV-positive cells was not altered by prenatal LPS experience. The red vertical dashed line and the red dashed boxes in the brain schemes show the position of the section, and analyzed structures, respectively. (**c**) Confocal fluorescence images of the secondary motor cortex labeled with PV (red) and WFL (green). Many PV-positive neurons are enwrapped by perineuronal nets, highlighting the close association between inhibitory neurons and extracellular matrix structures. Scale bar 250 µm. Grey boxes indicate the position of the detailed images. (**d**) Detailed images of PV-positive neurons with and without perineuronal nets at P28. The inset (grey box, lower right) shows WFL-positive, PV-negative cells. Additional blue staining by DAPI (blue) highlights cell nuclei. Scale bar 50 µm. Box plots show the median (horizontal line), first and third quartiles (lower and upper box boundaries), and whiskers extending to the minimum and maximum values within 1.5× the interquartile range. Dots represent individual data points. Number of animals per group: *n* = 7. *** *p* < 0.001; ** *p* < 0.01; * *p* < 0.05; # *p* = 0.05–0.10.

To quantify PV-positive interneurons and PNN in the prefrontal cortex (PFC), we used immunohistochemical staining. Prenatal treatment by LPS did not lead to changes in the number of PV-positive interneurons but elevated the number of cells with PNN (WFL-positive) (F(1, 12.5) = 5.307; *p* = 0.039) (Figure 9a). No significant changes in the number of PV-positive interneurons enwrapped by PNN (Figure 9a), nor in ratio of double-labeled cells to all PV-positive neurons (Figure 9b), suggest that association between inhibitory neurons and extracellular matrix structures was not disrupted by LPS experience. The significant effect of age showed that adult animals had lower number of PV-positive interneurons (F(1, 46) = 15.496; *p* < 0.001), as well as PV-positive interneurons enwrapped by PNN (F(1, 46) = 8.989; *p* = 0.004), than juveniles (Figure 9a). In addition, females exhibited a higher number of WFL-positive cells (F(1, 37.6) = 10.667; *p* = 0.002), especially at P28 (trend in interaction sex×age: F(1, 36.6) = 3.733; *p* = 0.061), compared to males (Figure 9a).

### 2.7. Prenatal LPS Decreased PV Protein Levels in Prefrontal Cortex

**Figure 10:**
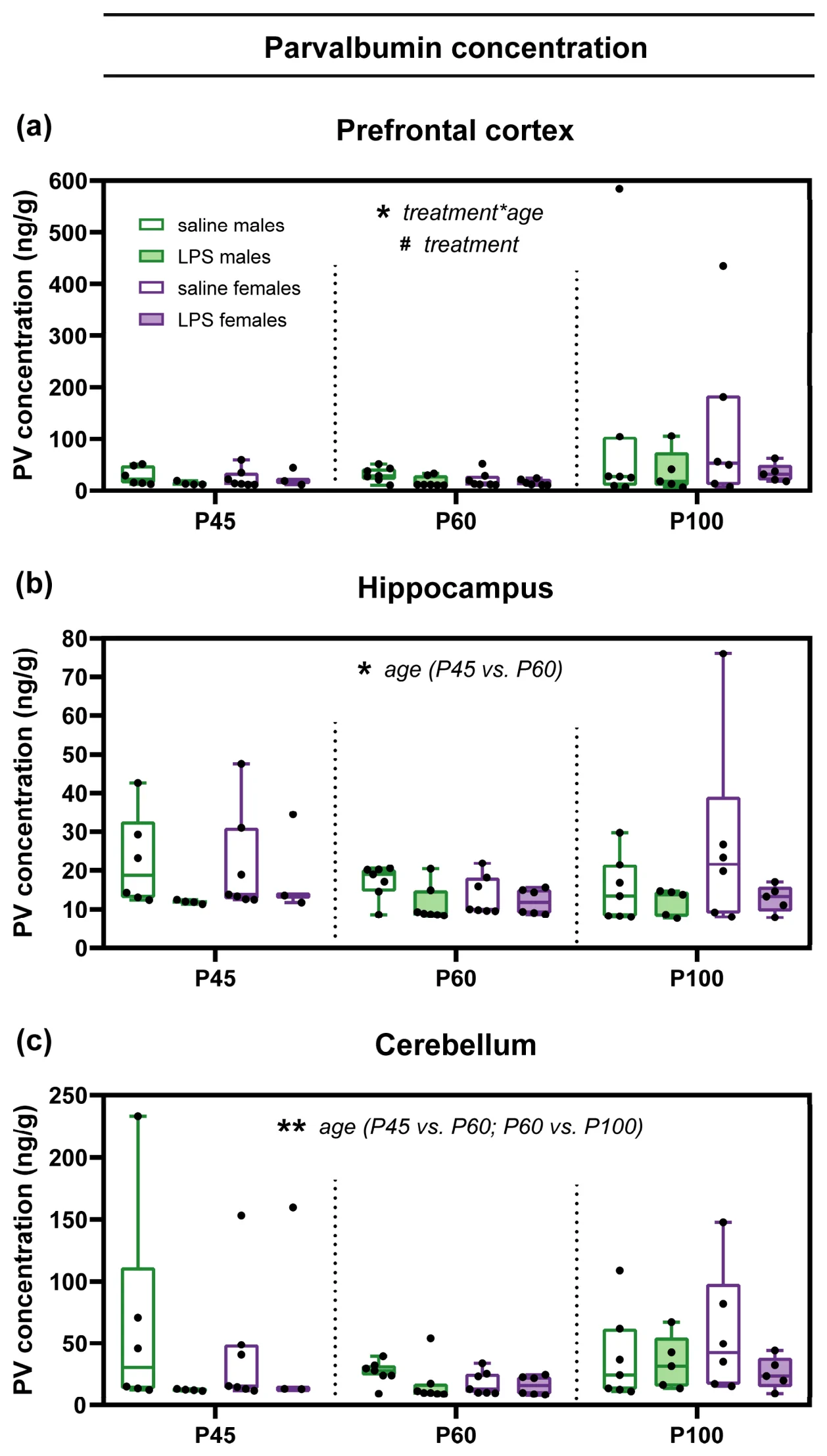
Parvalbumin (PV) protein levels in the prefrontal cortex, hippocampus and cerebellum at postnatal days P45, P60 and P100. Prenatal LPS treatment caused age-dependent decrease in PV levels in the prefrontal cortex (**a**) but not the hippocampus (**b**), and cerebellum (**c**). In addition, PV concentration was significantly lower at P100 compared to P60 and P45 in the cerebellum, and P60 to P45 in the hippocampus and cerebellum. Box plots show the median (horizontal line), first and third quartiles (lower and upper box boundaries), and whiskers extending to the minimum and maximum values within 1.5× the interquartile range. Dots represent individual data points. Number of animals per group: *n* = 3–7. ** *p* < 0.01; * *p* < 0.05; # *p* = 0.05–0.10.

PV protein concentrations in three different brain areas of adolescent and adult rats were quantified using Enzyme-Linked Immunosorbent Assay (ELISA). In the PFC, statistical evaluation revealed significant interaction treatment×age (F(2, 53.1) = 3.919; *p* = 0.026) and a trend in LPS treatment (F(1, 17.6) = 3.172; *p* = 0.092) showing that offspring of LPS-treated dams, compared with those of saline-treated dams, exhibited lower PV levels in the PFC depending on age (Figure 10a). However, prenatal LPS exposure did not lead to changes in PV levels in the hippocampus and cerebellum (Figure 10b,c). Only the significant effects of age (hippocampus: F(2, 51.2) = 4.576; *p* = 0.015; cerebellum: F(2, 51.2) = 5.147; *p* = 0.009) and post hoc tests revealed differences between ages P60 and P100 for cerebellum and P45 and P60 for both the hippocampus and cerebellum (Figure 10b,c). In addition, structural comparison revealed that the PFC showed the highest concentration of PV compared to the other two measured structures (F(2, 114.8) = 109.854; *p* < 0.001). This finding was somewhat unexpected, as studies in Wistar rats typically show highest PV levels in the cerebellum [23,24].

## 3. Discussion

We have examined the effect of LPS-induced MIA on brain and behavior of the PV-Cre Long-Evans rats at different ontogenetic timepoints, using the MIA protocol established by earlier studies [9,17,18].

We found that the PV protein levels in the PFC were quantitatively decreased in our model animals in an age-dependent manner. This contrasts with the existing literature, as MIA is not generally associated with changes in PV protein concentrations in the PFC, at least in rat models [25,26,27,28], whereas it is more common in mice [29,30,31,32,33]. However, we must note that none of the studies reporting PV protein levels used a comparable MIA protocol, making any comparisons problematic.

More often assessed is the number or density of PV-positive interneurons detected immunohistochemically. Surprisingly, our findings show no significant reduction in the number of PV-positive interneurons in the PFC. This is not necessarily in contradiction with the reduced PV protein levels mentioned above, as the concentrations of protein in a tissue and the number of PV-positive cells are two distinct measures [34,35]. However, this result contrasts with the usual findings from both patients with schizophrenia [36,37] and animal models of MIA [33,38]. Moreover, previous studies using the same MIA protocol, including our own [9,18], also found a decreased number of PV-positive interneurons.

The percentage of PV-positive interneurons surrounded by PNNs, in our case, was not altered by the LPS treatment. Even more surprisingly, we observed an overall increase in PNN numbers in the LPS group, which is unprecedented in MIA studies, where unchanged or decreased numbers of PNNs are the typical finding [39,40]. However, the fraction of double-labeled cells to the total PV-positive population was surprisingly low in the PV-Cre rats (including controls) at around 30%. There is considerable variability in this parameter among published studies: in Long-Evans rats, Jacobs-Brichford et al. reported numbers comparable to ours [41]; in other rat strains, the fraction ranges from about 45% to more than 70% [42,43], and up to 75% in mice [34]. Direct interstrain comparison of PNN densities might therefore deserve a dedicated future study.

Brain morphology patterns also differed from our previous findings, which showed general macrocephaly without altered hippocampal volume in Wistar juveniles after MIA [9]. In the PV-Cre Long-Evans rats, the total brain area at P28 was normal, the relative size of the hippocampus was not significantly changed after LPS exposure. Sex-dependent alteration of hippocampal volume had been previously reported in a rat poly(I:C) model [44,45], and several male-only studies reported reduced hippocampal volume after MIA [46,47].

Prenatal LPS exposure had no effect on the weight of the offspring, caused no deficits in sensorimotor functioning (apart from delayed righting response in P5 females) and only mild qualitative changes in pup USV, suggesting lack of gross developmental impairment. In older animals, the effects of treatment were also relatively few, not particularly strong, and almost universally in the form of statistical interactions with sex and/or age, making interpretations difficult.

Self-grooming patterns were altered after LPS treatment in both juveniles and adults. Grooming behavior is inherently heterogeneous, as it may represent displacement behavior elicited by the approach/avoidance conflict [48], de-arousal behavior following a stressful experience [49], or comfort behavior occurring when the animal feels safe, which makes it particularly difficult to interpret. It is considered independent on the traditional measures of anxiety, e.g., open arm exploration in the EPM [22,50]. Studies with anxiolytics provide an important insight into this ambivalence [51]: longer bouts of grooming with correct structure including tail grooming indicate comfort behavior, while short, unstructured and predominantly rostral grooming tends to be stress-related [52]. In our data, grooming events were mostly very short, with properly structured grooming bouts being nearly absent, which is more suggestive of the stress- or conflict-related types of grooming. Elevated self-grooming has been reported quite frequently after MIA [53,54,55,56], although generally as part of a more general affective phenotype, which was not demonstrated in our case.

Risk assessment in the elevated plus maze and initial exploration of the open field, which were also altered by the LPS treatment, are additional examples of behavior linked to motivational conflict. However, the LPS rats did not show unambiguous signs of anxiety, unlike our older study in Wistar rats [9]. In our case, eventual anxiogenic effects of the treatment might have been masked by elevated baseline anxiety in PV-Cre Long-Evans rats (relative to Wistar rats), despite regular handling. If the key behavioral outcome becomes very scarce even in the controls, as was the case with open-arm entries in the elevated plus maze and social play in juveniles (play is strongly suppressed by anxiety [57]), eventual anxiogenic effect of treatment may be undetectable due to floor effect.

In the present study, LPS females showed slightly increased locomotor activity, unlike in the previous study where adult LPS females were less active. Contrary to Vojtechova et al. [9] and established consensus for MIA models, there were no signs of sensorimotor gating impairments [8]. Social behavior and communication were slightly altered by the LPS treatment, but the pattern was not suggestive of a social deficit or withdrawal typical for models of ASD and schizophrenia. Ultrasonic communication was even enhanced by the LPS treatment. In general, the behavioral phenotype was very mild, and the model evidently lacked the core phenotypes of schizophrenia- or ASD-like behavior.

This led us to the question about the causes of this discrepancy. We have verified the immunogenicity of the LPS in an extra group of animals (Figure A1), because it was not practically possible to assess the immune reaction of the experimental pregnant dams. Neurodevelopmental consequences of MIA are very sensitive to context, such as animal handling, housing [58] or changes in the treatment protocol [59]. As our settings were essentially the same as in Vojtechova et al. [9], the genetic background (either strain and/or the transgenic status) was the most probable culprit. Rat strains differ in the extent and reliability of the cytokine response to immunological challenge [20], and strong interstrain differences in the impact of MIA on offspring have been noted in mice studies [19,60,61,62]. Although neurodevelopmental disorders have been previously observed after LPS-induced MIA in Long-Evans rats [63,64], the particular treatment regimen used here has not been tested in Long-Evans rats so far. It is also possible that Wistar and Long-Evans rats differ in baseline parameters at the molecular and cellular level (e.g., PV expression, PNN density, etc.). Only a direct comparative study of the two strains could settle this issue.

Our present study may serve as a cautionary tale, highlighting the importance of genetic background in the phenotypic manifestation of a MIA model. Our original intention was to use the PV-Cre transgenic rats for targeted manipulations of PV-positive interneurons, to examine their mechanistic role in MIA-induced behavioral alterations. However, given the rather mild consequences of MIA in the PV-Cre Long-Evans rats compared to the Wistar rats, especially in the crucial domains such as social behavior or sensorimotor gating, we had to abandon that goal altogether. The degree to which the choice of a rat strain/line influences the phenotype is itself an important finding. Further research of the strain effects on the extent of immune activation and its impact upon the offspring is certainly recommended, as it may elucidate the genetic mechanisms of vulnerability and resilience governing the course of neurodevelopmental disorders, including schizophrenia and ASD.

## 4. Materials and Methods

### 4.1. Animals

Transgenic PV-Cre Long-Evans rats (HsdSage:LE-Pvalb*^em1(IRES-Cre)Sage^*) were purchased from Envigo (now Inotiv, Inc., West Lafayette, IN, USA) via AnLab (Vídeňská 1083, Prague 4, Czech Republic). Note that the CRE recombinase was not used in the presented experiments, the strain was used to enable follow-up chemogenetic manipulation experiments in the MIA model.

In total, 31 experimental litters of PV-Cre Long-Evans rats (375 offspring in total) from dams undergoing either MIA (13 litters) or the control procedure (18 litters) were used in the study. Selected individuals from all the litters were used for brain tissue sampling at various timepoints. Out of the total number, 72 individuals from 15 litters (8 MIA, 7 control) were used for longitudinal behavioral experiments (from pup to adult). No more than 4 pups of each sex from the same litter were kept in the same group to ensure a balanced design.

The rats were kept at the National Institute of Mental Health (NIMH-CZ) in Klecany, Czech Republic, in an air-conditioned room with a 12 h/12 h light/dark cycle and food and water ad libitum. Pregnant females were separated several days before delivery into larger breeding boxes (55 × 32 cm) with available nesting material, where they remained with the offspring until weaning. Experimental animals were housed in standard Plexiglas boxes (46 × 24 cm) in groups of 2 or 3 same-sex siblings, with wooden blocks and plastic tunnels for enrichment. All procedures and manipulations took place during the light phase of the daily cycle.

All experiments were approved by the Institutional Animal Care and Use Committee at NIMH (Project of Experiment No. 81) and by the Departmental Expert Committee for the Approval of Projects of Experiments on Animals of the Ministry of Health of the Czech Republic (Project of Experiment No. 21/2023) and complied with the Animal Protection Act of the Czech Republic, EU directive (2010/63/EU).

### 4.2. LPS Verification and Determination of Serum Nitrite/Nitrate Levels

To verify the ability of bacterial LPS used in our study (*Escherichia coli*; L-3755, Serotype 026:B6; Sigma-Aldrich/Merck, Prague, Czech Republic) to trigger an immune reaction (specifically, the production of nitric oxide as a proxy of the systemic inflammation), we determined nitrite/nitrate (NO^2−^/NO^3−^) levels in a blood serum of C57BL/6J mice and PV-Cre Long-Evans rats. LPS is a potent inducer of nitric oxide (NO) production. NO is subsequently oxidized to nitrite and nitrate, which can therefore be quantified as indirect indicators of systemic NO production and inflammatory activation.

The blood serum was collected 6 h after the intraperitoneal LPS administration (5 mg/kg for mice; 1 mg/kg for rats), centrifuged for 15 min at 1520 RCF, 10 °C, and stored at −80 °C for later analysis.

Serum samples were deproteinated by filtration through Amicon Ultra centrifugal filter units (Merck, Darmstadt, Germany; 10 kDa molecular weight cut-off). The concentration of nitrite/nitrate, oxidation products of nitric oxide, was determined using a Nitrite/Nitrate Assay Kit (Merck, #23479). Briefly, undiluted samples were incubated with nitrate reductase and the provided cofactors for 2 h at 25 °C, converting nitrate to nitrite. Total nitrite was then quantified using the Griess reaction, with absorbance measured at 540 nm. Concentrations were calculated from a sodium nitrite standard curve.

### 4.3. MIA Model Preparation

After 5 days of handling and weighing the breeding rat females, we determined the estrous cycle using the protocol by [65]; for details see our previous work Vojtechova et al. [9]. Females in the estrus or proestrus were mated with a male for 24 h. The day of the mating was defined as gestational day (GD) 0. Pregnant rat females received 8 subcutaneous (s.c.) injections of bacterial LPS from *Escherichia coli* (L-3755, Serotype 026:B6; Sigma-Aldrich/Merck, Prague, Czech Republic) at a dose of 1 mg/kg and injection volume of 1 mL/kg, which was administered every other day from the GD7 to GD21 (delivery), according to Basta-Kaim et al. [17,18] and Vojtechova et al. [9]. The LPS was dissolved in 0.9% saline solution, the vehicle alone served as a control treatment.

### 4.4. Postnatal Manipulations and Behavioral Testing of the Offspring

Four days after delivery, the dams with pups were left undisturbed. The pups were sexed at P5 and underwent weighing and simple behavioral tests at P5, P7, P10, P20, P21, P26 and P27. They were weaned at P28. During the testing battery, the dam was separated from the pups for no longer than 30 min, the breeding box with neonates was placed on a heating pad and the pups were replaced into the nest between individual tests to maintain body temperature. Males were tested before females from each litter. Subsequently, the offspring was tested in behavioral tasks at P45, P90 and P110. All adolescent and adult males always underwent the particular session before all females, and apparatuses were thoroughly cleaned with water and denatured alcohol mix to eliminate residual odors. See the timeline of experiments in Figure 1.

#### 4.4.1. Audio and Video Recording

Behavioral procedures were video recorded by an overhead digital camera (LogiTech Webcam C920; Logitech International, Lausanne, Switzerland) at 30 fps. Neonatal USV was recorded by the Ultramic 250 K microphone (Dodotronic, Castel Gandolfo, Italy) at a sampling frequency of 250 kHz and analyzed by USVSEG [66].

#### 4.4.2. Righting Reflex (Pups)

At P5 and P7, the pups were tested for reflex development [67,68]. Each rat pup was removed from the homecage, gently placed onto a flat rubber pad in supine position and then released. The time it took them to turn over to the prone position (when both hindlimbs touched the pad) was measured. The test was repeated twice, and the better result was taken as representative.

#### 4.4.3. Neonatal Ultrasonic Vocalization (Pups)

At P7 and P10, we assessed the social distress USV of the pups isolated from their mothers. Each pup was removed from the nest and placed into a small box (30 × 18 × 14.5 cm) with clean dust-free bedding and kept undisturbed. Isolation-induced USV was recorded for 60 s. The measured parameters were the total number of USV, the total duration of USV, the maximal frequency (call frequency at the moment of the highest amplitude), the cv frequency (coefficient of variation of peak frequencies, quantifying the degree of frequency modulation of each syllable) and the maximal amplitude (peak loudness, in dB) (Figure 2).

#### 4.4.4. Homing Task (Pups)

At P7, we evaluated the motivation and ability of the pups to recognize the odor of their home nest and preferentially navigate to it. A pup was placed into a box (30 × 18 cm) separated into two halves: one was filled with clean bedding, the other with bedding from the homecage. The divider separating the compartments enabled free movement of the animal but was visible from above. The pup was positioned perpendicularly to the divider with the head in the clean half, and the base of the tail on the dividing line. The trial was counted as successful when the pup crossed the divider with the whole body and both hindlimbs within 60 s, and the time was measured. Each rat was assigned to two trials and the better result was taken as representative.

#### 4.4.5. Negative Geotaxis (Pups)

At P7 and P10, we examined sensorimotor development and an automatic response to gravitational stimuli in the pup offspring [69]. Each pup was placed in a prone position head down on a 30° inclined surface covered with rough rubber. The latency to rotate 180° and head upslope was measured, with a maximum time of 60 s. The test was repeated twice, and the better result was taken as representative.

#### 4.4.6. Bar Holding (Pups)

At P7 and P10, we evaluated sensorimotor coordination and grip strength in pups [67,70]. A pup was put on a grooved wooden horizontal rod (65 cm length, 8 mm in diameter) and was allowed to reflexively grasp the rod by its forepaws and hang as long as possible (maximum time of the trial 60 s). Time to fall into a box filled with soft towels was recorded. All pups were tested twice with at least 30 s of resting time between the trials; the better result was taken as representative.

#### 4.4.7. Open Field (Juveniles)

At P20 and P26, juvenile rats underwent the OF test which also served as adaptation to the environment before the social play test. Each rat alone freely explored an unknown and dimly illuminated (18 lux) plexiglass box (36 × 33 × 18 cm) filled with clean dust-free bedding for 5 min. Bedding was the same for all individuals from one litter, but each litter had its own box and bedding (note that the animals were not weaned yet and used to the communal scent). Rat behavior was recorded by an overhead camera, and recorded videos were analyzed offline automatically in the EthoVision XT 16 software (Noldus Information Technology, Wageningen, The Netherlands). The total walked distance was used as a measure of locomotor activity. The time spent in the center of the OF (the center represented the equal area as the peripheral part (Figure 4)) and frequency of center visits were considered a measure of anxiety.

#### 4.4.8. Social Play (Juveniles)

At P21 and P27, we explored the social behavior of juveniles [71]. After 5 min social isolation, two rats from the same litter and sex were put into a box simultaneously and left undisturbed for 5 min (only the first 3 min of interaction were analyzed). The social test was performed in the same box, with the same bedding and illumination as the OF test performed the day before. We evaluated social play, other social contact (e.g., mutual sniffing and touching), and self-grooming and digging in the bedding as potentially repetitive behavior (see the example snapshots in Figure 4). Recorded videos were analyzed offline manually using the BORIS software (v. 8.27.1) [72] by a blinded observer; each animal from the pair was scored separately.

#### 4.4.9. Elevated Plus Maze (Adolescents, Adults)

At P45 and P90, we assessed anxiety and activity behaviors in the EPM [71,73]. Both adolescents and adults were tested in the identical cross-shaped apparatus made from light gray plastic. The apparatus was elevated 74 cm above the floor and consisted of four arms (50 cm long, 10 cm wide). Two opposite arms were surrounded by 30 cm high walls (“closed”; illumination 30 lux; safer for the animals), the other two did not have walls (“open”; illumination 70 lux). Each rat was placed into the central part (“center”) with its head in an open arm and left to freely explore the apparatus for 5 min. Rat behavior was recorded by an overhead camera, and recorded videos were analyzed offline in the BORIS software [72] by a blinded observer. We evaluated frequency and time spent in the open arms and defecation as measures of anxiety (a visit of the arm was counted when the whole body was in), time spent in the center, and looking down from the maze (when the head of the animal protruded over the edge of an open arm) as a form of risk assessment behavior, and self-grooming as a potentially stereotyped behavior (see the example snapshots in Figure 5).

#### 4.4.10. Open Field (Adolescents, Adults)

At P45 and P90, spontaneous locomotor activity and anxiety-related behaviors of adolescent and adult rats were measured in a square white-laminated chipboard OF arena (70 × 70 cm, 40 cm high walls, illumination 800 lux) with an overhead camera recording rat behavior [9,71]. Each rat was placed into the middle part and left to freely explore the arena for 10 min. Spontaneous locomotor activity (total walked distance) and anxiety (time spent in the center of the arena) were assessed using the EthoVision software (Noldus Information Technology, Wageningen, The Netherlands). The areas of the central and peripheral part of the arena were equal (Figure 5). The sessions were analyzed as a whole (“total”) or divided into two parts (“0–5 min” and “5–10 min”). The OF session also served as a habituation to the arena used in the SI test performed a day later. Both adolescents and adults were tested in the identical apparatus.

#### 4.4.11. Social Interaction and Ultrasonic Vocalization (Adolescents, Adults)

At P45 and P90, social behavior and communication were evaluated in the simple SI test in a neutral environment; USV was recorded during social sessions [9,71]. First, two unfamiliar rats (non-cage mates) of the same sex and from the same treatment group underwent social isolation (10 min). After that, they were simultaneously placed in opposite corners of the dimly illuminated (14 lux) OF arena, already familiar to them, facing the walls and left to interact for 10 min (only the first 5 min of interaction were analyzed). Videos recorded by an overhead camera were analyzed offline manually using the BORIS software [72] by a blinded observer. We assessed the following parameters: (a) social behavior: anogenital exploration (nose contact with the anogenital area or tail base of the partner), social contact (sniffing or touching the other rat’s body except the anogenital area or tail), following the social partner, climbing over the social partner, play/fight, and evading; and (b) non-social and potentially repetitive behavior [74]: self-grooming and freezing. Behaviors which were too rare for meaningful analysis (climbing over the social partner, play/fight, evading and freezing) are not reported. Each animal from the pair was scored separately. The identical pairs of animals were tested as adolescents and again in adulthood.

#### 4.4.12. Y-Maze (Adolescents, Adults)

At P45 and P90, spontaneous alternation as a measure of working memory was tested in the Y-maze task [71]. For both adolescents and adults, we used a dimly illuminated (25 lux) three-armed maze made of light gray plastic. The arms were marked “A”, “B” and “C” and were identical (50 cm long, 10 cm wide, 30 cm high walls). Each rat was placed at the end of the “A” arm facing the center of the maze. The session of free exploration lasted 8 min. The order of visits of individual arms was noted from the video recording. We calculated spontaneous alternation (in %) as the number of correct triads of arm entries (ABC, ACB, BCA, BAC, CAB, CBA) divided by the number of all triads. Repeated visits of the same arm (AA, BB, CC) were counted as incorrect. Arm entrance was counted when all four limbs of the rat were inside. Total arm visits were used to show general activity of the animal.

#### 4.4.13. Beam Walking (Adolescents, Adults)

At P45 and P90, animals underwent a beam-walking test assessing sensorimotor coordination and locomotor abilities. Each rat had to walk along a wooden beam (2 m long), at first wide (4.5 cm) and then narrow (2.2 cm), elevated 1 m above the floor [9,75]. The distance between the start (closed by a barrier) and the goal (homecage) at the opposite end varied between 0.5 m, 1 m and 2 m (see Table 1). The rats were released from 1/4 and 1/2 of the beam length (Training) before full-length trials (Test) to familiarize them with the task. Only the full-length trials were evaluated. At the beginning of each trial, a rat was placed on the start end of the beam facing the goal and had to reach an empty homecage; the cage-mate was waiting in a closed cage nearby. The performance of rats was watched by two experimenters from opposite sides. Maximum trial time was 2 min. The measured parameters were the time needed to traverse the beam and the number of errors, i.e., the number of footslips (when one or more of the rat’s paws slipped the upper surface of the beam), the number of near-falls (severe loss of balance without fall) and the number of falls. Error score was calculated as the total number of footslips, with near-fall scoring as five slips and a fall scoring as 10 slips. Both adolescents and adults walked on identical apparatus using the same protocol.

#### 4.4.14. Prepulse Inhibition (Adults)

At P110, rats were evaluated for sensorimotor gating in a PPI task [9,76]. PPI occurs when a preceding shorter and weaker acoustic stimulus (prepulse) physiologically inhibits the startle reaction to a stronger acoustic stimulus (pulse). The startle reflex was measured as a jumping reaction to acoustic stimuli in rats closed in a transparent tight tube enclosed in a soundproofed box (SR-LAB Startle Response System, San Diego Instruments, San Diego, CA, USA). The session started with habituation to white noise (70 dB, 5 min). Subsequently, the rat was exposed to 50 trials with acoustic stimuli in a pseudorandom order and irregular inter-trial intervals (12–20 s). The trials included a strong pulse alone (120 dB; duration 20 ms) or preceded by prepulse (3, 5, or 10 dB above the white noise level, i.e., 73, 75, or 80 dB; duration 20 ms; interval between prepulse and pulse 100 ms), or control condition with no stimulus. PPI (shown in %) was calculated as a relative decrease in the startle reaction by the prepulse, according to the formula:

[PPI = 100 − (PP/P120) × 100]

where PP represents the startle reaction to a pulse with a prepulse, while P120 represents the startle reaction to a pulse without a prepulse. Because of the stressful nature of the task, it was done only in the adults as the very last behavioral test.

### 4.5. Immunohistochemistry

#### 4.5.1. Brain Tissue Preparation

Rats (P28, P110) were deeply anesthetized by xylazine (30 mg/kg) and ketamine (200 mg/kg) and transcardially perfused with 0.9% saline, followed by 4% paraformaldehyde in 0.1 M phosphate buffer (pH 7.4). Brains were then removed and post-fixed overnight at 4 °C in 4% paraformaldehyde prepared in 0.1 M phosphate-buffered saline (PBS), and subsequently cryoprotected in 30% sucrose until they sank. Coronal brain sections (50 µm thick; one-in-five series) were cut using a cryostat (Leica CM1860 UV). For histological and immunohistochemical analyses, all sections were mounted on gelatin-coated slides.

#### 4.5.2. Morphology Analysis

For general morphological analyses, toluidine blue staining was performed using a 1% solution of toluidine blue powder (Biognost, Zagreb, Croatia, catalog# TB-P-25) prepared in 70% ethanol. Brain sections were rinsed with distilled water and incubated in the toluidine blue solution for 7 min. Afterward, slices were extensively rinsed with distilled water, differentiated in 60% and 100% ethanol, cleared in NeoClear for 10 min (Merck, catalog# 109843). Subsequently, sections were mounted on slides and coverslipped with NeoMount (Merck, catalog# 109016). Tile-scan images were acquired using a confocal microscope Leica TCS SP8X (Leica Microsystems, Mannheim, Germany) equipped with a 5× objective (NA 0.15; 512 × 512 pixels). The area of the dorsal hippocampus was measured at approximately −2.4–−3.4 mm from bregma, according to the Paxinos and Watson atlas [77]. Measurements were performed manually using custom software developed in-house specifically for this purpose. Five sections per rat were analyzed, and hippocampal areas were expressed as a percentage of the total brain area.

#### 4.5.3. Parvalbumin and Perineuronal Nets Analysis

Non-specific binding was blocked with 10% normal donkey serum (Merck, catalog #S30-M) and 0.3% Triton X-100 for 1 h at room temperature. Free-floating brain sections were incubated with primary antibodies diluted in 0.1 M PBS containing 0.3% Triton X-100 overnight at 4 °C. For PNN analysis, sections were incubated with Wisteria floribunda lectin (WFL) conjugated to fluorescein (1:1000; ThermoFisher, Fisher Scientific, Pardubice, Czech Republic, catalog #L32481) and a mouse monoclonal anti-PV antibody (1:3000; Sigma-Aldrich/Merck, Prague, Czech Republic, catalog #P3088). After primary antibody incubation, sections were rinsed in 0.1 M PBS and incubated with secondary antibody in 0.1 M PBS containing 0.3% Triton X-100 for 2 h at RT. Secondary antibody conjugated to Alexa Fluor 594 (donkey; 1:500; Jackson ImmunoResearch, West Grove, PA, USA, catalog #715-585-151) was used. Sections were rinsed in 0.1 M PBS and coverslipped with ProLong Gold Antifade Reagent with DAPI (Cell Signaling Technology, Leiden, Netherlands, catalog #8961). Confocal images were acquired using a Leica TCS SP8X confocal microscope (Leica Microsystems, Mannheim, Germany) equipped with a 10× objective (NA 0.4). Fluorophores were excited using 488 nm and 594 nm laser lines, and emission was detected using appropriate spectral detection settings. Z-stacks were acquired with a step size of 2.0 µm (6 optical sections per stack). Images were collected using the Leica Application Suite X, 3.5.7. 23225. Image processing and analysis were performed using the ImageJ software (version 1.47, NIH, Madison, WI, USA). Maximum-intensity projections were generated from z-stacks for visualization; brightness and contrast adjustments were applied uniformly across all images. For each animal, three coronal sections from the left and right hemispheres were analyzed separately at matched rostrocaudal levels, +4.2 mm from bregma, according to the Paxinos and Watson atlas [77]. Quantification was performed in the PFC, specifically within the secondary motor cortex (M2) and prelimbic cortex (PrL). The number of parvalbumin-positive (PV-positive) and Wisteria floribunda lectin–positive (WFL-positive) cells was quantified in each region. Cell counts were obtained separately for the left and right hemispheres.

### 4.6. Parvalbumin Protein Levels

Rats (P45, P60, P100) were decapitated under isoflurane anesthesia, their brains quickly removed and briefly washed in ice-cold saline solution. The brain was then quickly dissected, and the regions of interest (PFC, hippocampus and cerebellum) were placed into sterile Eppendorf tubes and flash-frozen on dry ice and stored at −80 °C. The PFC was sampled by slicing the frontal pole of the hemisphere at a 45° angle to the coronal and horizontal plane, and care was taken to avoid the underlying white matter.

Rat parvalbumin concentrations in the samples were quantified using a commercial sandwich ELISA kit (Antibodies.com. Stockholm, Sweden, catalog #A5671) according to the manufacturer’s instructions. This assay operates within a dynamic range of 0.781–50 ng/mL with a minimum detection sensitivity of 0.31 ng/mL.

Prior to homogenization, tissues were rinsed thoroughly in ice-cold PBS (pH 7.4) and weighed. Tissues were minced and homogenized on ice in PBS at a ratio of 1 g tissue to 9 mL PBS. Cells were further disrupted via ultrasonic lysis or freeze–thaw cycling. Homogenates were centrifuged at 5000× *g* for 5 min, and the resulting supernatant was stored at −80 °C for analysis.

### 4.7. Statistical Analysis

For LPS verification, we used the GraphPad Prism software (version 8.0.1). Two-way Analysis of Variance (ANOVA) with treatment (LPS or saline) and species (mice and rats) as between-subject factors was used. Holm–Šidák’s multiple comparisons test was used to confirm the effect in both rodent species.

Statistical analyses of other experiments were performed in R (version 4.2.2; R Core Team, 2022). For each outcome, the analysis method was selected based on the assumption checks: outcomes meeting parametric assumptions were analyzed with linear mixed models (LMM), the remaining outcomes with Aligned Rank Transform (ART) ANOVA. Both approaches used identical fixed and random effect structures: treatment (LPS vs. saline), sex (males vs. females), age and all their interactions as fixed factors, and random intercepts for run, litter, subject (individual animal accounting for repeated measures) and pair dependency of animals in social interactions. Effects of sex and age were reported only in selected parameters. See the detailed description of statistical analysis in the Supplementary Material.

We accepted significance at *p* ≤ 0.05. The *p*-values between 0.05 and 0.1 were considered as trends.

## 5. Conclusions

To sum up, chronic LPS treatment in the PV-Cre Long-Evans rat induced only very subtle and often sex-dependent behavioral changes in the offspring, which could be attributed to disturbed neurodevelopment, but no specific signs of either schizophrenia or ASD. Examinations of the brains showed the expected decrement of PV protein in the prefrontal cortex, but no decrease in PV-positive interneuron numbers and even a paradoxical increase in PNN counts in the LPS offspring. We have to conclude that in the PV-Cre Long-Evans rats, our MIA protocol did not elicit the same schizophrenia- or ASD-like alterations as described by Basta Kaim et al. [17] and Vojtechova et al. [9] in the Wistar rats. Our results suggest that genetic background may influence the manifestation of MIA-induced phenotype, highlighting the crucial role of animal strain choice in the study of neurodevelopmental disruptions.

## Figures and Tables

**Table 1:** Beam-walking test design.

	Training		Test (Wide)		Training	Test (Narrow)			
Trial	1	2	3	4	5	6	7	8	9
Width	4.5 cm	4.5 cm	4.5 cm	4.5 cm	2.2 cm	2.2 cm	2.2 cm	2.2 cm	2.2 cm
Length	0.5 m	1 m	2 m	2 m	1 m	2 m	2 m	2 m	2 m

## Data Availability

The raw data supporting the conclusions of this article will be made available by the authors on request.

## Supplementary Materials

The following supporting information can be downloaded at: https://www.mdpi.com/article/10.3390/ijms27219649/s1, [78,79,80,81,82,83,84,85,86].

## Abbreviations

The following abbreviations are used in this manuscript:

ANOVA	Analysis of variance
ASD	Autism spectrum disorder
ELISA	Enzyme-Linked Immunosorbent Assay
EPM	Elevated plus maze
GD	Gestational day
IL	Interleukin
LPS	Lipopolysaccharide
M2	Secondary motor cortex
MIA	Maternal immune activation
OF	Open field
P	Postnatal day
PFC	Prefrontal cortex
polyI:C	Polyinosinic:polycytidylic acid
PBS	Phosphate-buffered saline
PNN	Perineuronal net
PPI	Prepulse inhibition
PrL	Prelimbic cortex
PV	Parvalbumin
s.c.	Subcutaneous
SI	Social interaction
TLR4	Toll-like receptor 4
TNF	Tumor necrosis factor
USV	Ultrasonic vocalization
WFL	Wisteria floribunda lectin
