# Supplementary material for: Maternal Immune Activation by Lipopolysaccharide Reduced Parvalbumin Levels and Increased Perineuronal Nets in the Prefrontal Cortex Without Inducing Schizophrenia-like Behavioral Deficits in PV-Cre Long-Evans Rats
Source: Int J Mol Sci. 2026 Oct 28;27(21):9649. doi: 10.3390/ijms27219649 (PMC13763192; doi:10.3390/ijms27219649)
Supplement: Supplementary file 1 [file ijms-27-09649-s001.zip › ijms-4588160-supplementary.pdf]

Supplemental Material

Maternal Immune Activation by Lipopolysaccharide Reduced Parvalbumin Levels and Increased Perineuronal Nets in the Prefrontal Cortex Without Inducing Schizophrenia-like Behavioral Deficits in PV-Cre Long-Evans Rats

Iveta Vojtechova [1], Klara Tuckova [1,2], Viera Kutna [1], Barbora Jelinkova [1,3], Katerina Skalova [1,2], Maria Lebedeva [1], Anastasiia Khodak [1,2], Tomas Machacek [4] and Tomas Petrasek [1,*]

1 National Institute of Mental Health, Topolova 748, 25067 Klecany, Czech Republic;
iveta.vojtechova@nudz.cz (I.V.); klara.tuckova@nudz.cz (K.T.); viera.kutna@nudz.cz (V.K.); barbora.jelinkova@nudz.cz (B.J.); katerina.skalova@nudz.cz (K.S.); mariia.lebedeva@nudz.cz (M.L.); anastasiia.khodak@nudz.cz (A.K.)
2 Department of Physiology, Faculty of Science, Charles University, Albertov 6, 12800 Prague 2, Czech Republic
3 Department of Zoology, Faculty of Science, Charles University, Albertov 6, 12800 Prague 2, Czech Republic
4 Department of Parasitology, Faculty of Science, Charles University, Albertov 6, 12800 Prague 2, Czech Republic;
tomas.machacek@natur.cuni.cz
* Correspondence: tomas.petrasek@nudz.cz

Statistical analysis

Statistical analyses were performed in the R software (version 4.2.2; R Core Team, 2022).

For each outcome, the analysis method was selected based on the assumption checks described below: outcomes meeting parametric assumptions were analyzed with linear mixed models (LMM), the remaining outcomes with the Aligned Rank Transform (ART) ANOVA. Both approaches used identical fixed- and random-effect structures.

Pup tests and ART ANOVA

Pup developmental tests were analyzed separately for each test. Righting reflex (P5, P7), negative geotaxis (P7, P10) and bar holding (P7, P10) were each measured twice in the same animals. Homing was measured once (P7).

Before the main analysis, the distribution of each outcome was assessed separately for each age group. We used the Shapiro–Wilk test on raw values and on residuals after removing treatment × sex group means, skewness, excess kurtosis, the number of extreme values (beyond 3 × IQR) and the Fligner–Killeen test for homogeneity of variance between groups.

In negative geotaxis, animals that did not complete the test were given the maximum latency of 60 seconds, which produced a ceiling effect. All outcomes deviated markedly from normality: residuals were non-normal (Shapiro–Wilk $p < 0.05$), distributions were right-skewed with heavy tails, and some had ceiling values or unequal variances. The parametric assumptions of ANOVA and linear mixed models were therefore not met, and the data were analyzed with the non-parametric ART ANOVA (Wobbrock et al., 2011) using the ARTool package (Kay et al., 2025).

Because all age groups of a specific behavioral test were analyzed in a single model, the same method was used for the whole test even in cases where an individual age group did not violate normality (as in righting reflex P5).

The ART procedure aligns the response variable for each fixed effect and interaction, ranks the aligned values, and fits a separate model on the ranks for each effect. To account for the hierarchical structure of the data, the models were fitted as linear mixed models (lme4; Bates et al., 2015) with random intercepts: subject in

behavioral tests with repeated measures, litter and run in other behavioral tests. Pups were not independent observations: treatment was assigned at the litter level, pups from the same litter share genetic and maternal influences, and litters were tested in three consecutive experimental runs.
For righting reflex, negative geotaxis and bar holding, the model included treatment (LPS vs. saline), sex (males vs. females) and age as fixed factors with all their interactions. Random intercepts were included for run, litter and subject (individual animal accounting for repeated measures):

For righting reflex, negative geotaxis and bar holding, the model included treatment (LPS vs. saline), sex (males vs. females) and age as fixed factors with all their interactions. Random intercepts were included for run, litter and subject (individual animal accounting for repeated measures):

$$response \sim Age \times Treatment \times Sex + (1 \mid Run) + (1 \mid Litter) + (1 \mid Animal)$$

Homing was measured at one timepoint only (7 days of age), so the model included treatment and sex as fixed factors with their interaction, and random intercepts for run and litter:

$$response \sim Treatment \times Sex + (1 \mid Run) + (1 \mid Litter)$$

Litter identifiers were unique across runs, so litters were nested within runs. Significance of fixed effects was tested using Type III Wald F tests with KenwardóRoger approximated degrees of freedom (Kenward and Roger, 1997). Significant main effects and interactions were followed by post hoc pairwise comparisons using the ART-C procedure (Elkin et al., 2021), with p-values adjusted for multiple comparisons by the Benjaminió Hochberg false discovery rate method (Benjamini and Hochberg, 1995). Statistical significance was set at $\alpha = 0.05$.

The study included 72 pups from 15 litters including 36 LPS vs. 36 saline and 36 males vs. 36 females tested in 3 experimental runs. Time output for righting reflex at 5 days of age was not obtained in 24 pups because data from one of the runs were lost and these observations were treated as missing. The mixed model used all available data from the remaining animals.

LMM

Outcomes that met the assumptions of parametric analysis (normally distributed residuals, no ceiling or floor effects, no extreme outliers and homogeneous variances between groups) were analyzed with LMM fitted by restricted maximum likelihood using the lme4 and lmerTest packages (Bates et al., 2015; Kuznetsova et al., 2017). The fixed and random structure was identical to the ART models: treatment, sex, age and all their interactions as fixed factors, and random intercepts for run, litter and individual animal ($response \sim Age \times Treatment \times Sex + (1 \mid Run) + (1 \mid Litter) + (1 \mid Animal)$). Sum-to-zero contrasts were used, and fixed effects were tested with Type III F tests with KenwardóRoger approximated degrees of freedom (Kenward & Roger, 1997). Significant effects were followed by pairwise comparisons of estimated marginal means (emmeans package; Lenth and Piaskowski, 2026) with BenjaminióHochberg correction (Benjamini and Hochberg, 1995). Model assumptions were verified by inspecting residual QóQ plots and residuals-versus-fitted plots and by the ShapiroóWilk test on model residuals.

Ultrasonic vocalizations in pups

Pup ultrasonic vocalizations were recorded at P7 and P10, and five parameters were analyzed: total number of calls, total call duration (ms), maximal frequency (kHz), coefficient of variation of frequency, and maximal amplitude (dB). The analysis method was selected separately for each parameter based on the results of descriptive statistics (QóQ plots of residuals, ShapiroóWilk test, skewness, kurtosis and FligneróKilleen test). Call number, call duration, maximum frequency and frequency variation deviated from normality and were analyzed with ART ANOVA, whereas maximum amplitude met the parametric assumptions and was analyzed

with LMM. Both approaches used the same model structure, with treatment, sex and age as fixed factors and random intercepts for run, litter and animal (*parameter ~ Age × Treatment × Sex +(1 | Run)+(1 | Litter)+(1 | Animal)*). Significant effects were followed by pairwise comparisons (ART-C contrasts or estimated marginal means, respectively) with Benjamini–Hochberg correction.

Weight data

Animal weights at 9 selected timepoints were analyzed using LMM.

Juvenile, adolescent and adult tests

For each outcome, the analysis method was selected based on the assumption checks: outcomes meeting parametric assumptions, which was the juvenile open field test, locomotor activity in the adolescent/adult open field test and alternation ratio in Y-maze, were analyzed with linear mixed models (LMM); the remaining outcomes (social play, elevated plus maze, center duration in the adolescent/adult open field test, adolescent/adult social interactions, arm entrances in Y-maze, beam walking, prepulse inhibition) were analyzed with the Aligned Rank Transform (ART) ANOVA as described above. Both approaches used identical fixed-and random-effect structures: treatment (LPS vs. saline), sex (males vs. females), age and all their interactions as fixed factors, and random intercepts for run, litter and subject (individual animal accounting for repeated measures). Pair dependency of animals tested in pairs in social interactions test and social play test was accounted for by including a random intercept for pair in the mixed model, in addition to random intercepts for run, litter and subject.

Multiplicity was addressed at two levels. First, post hoc pairwise comparisons were performed only for effects that were significant in the omnibus test (ART ANOVA or linear mixed model, $p < 0.05$). Second, all post hoc p-values were adjusted using the Benjamini–Hochberg false discovery rate procedure.

The data were analyzed separately for each test. Juvenile tests (open field and social play) and adolescent/adult tests (elevated plus maze, open field, social interactions, Y-maze, beam walking) comprised two measurements in the same group of animals (P21 and P27 or P45 and P90, respectively). Prepulse inhibition was only measured once (P110).

Before the main analysis, the distribution of each outcome was assessed separately for each age group. We used the Shapiro–Wilk test on raw values and on residuals after removing treatment × sex group means, skewness, excess kurtosis, the number of extreme values (beyond 3 × IQR) and the Fligner–Killeen test for homogeneity of variance between groups.

Histology and ELISA

The parametric assumptions were met by the brain area and relative area of the hippocampus on brain slices, as well as numbers of PV-positive cells, WFL-positive cells and double-stained cells. The ART ANOVA was used for the ratio of double-labeled cells to all PV-positive neurons, as well as PV levels measured by ELISA.

Both approaches used identical fixed- and random-effect structures: treatment (LPS vs. saline), sex (males vs. females), age and all their interactions as fixed factors, and random intercepts for run and litter. Repeated measures did not apply for the parameters measured from post-mortem tissue

References

- Bates D, Mächler M, Bolker B, Walker S (2015). Fitting linear mixed-effects models using lme4. *Journal of Statistical Software* 67(1): 1ó48. DOI: 10.18637/jss.v067.i01
- Benjamini Y, Hochberg Y (1995). Controlling the false discovery rate: a practical and powerful approach to multiple testing. *Journal of the Royal Statistical Society B* 57: 289ó300. DOI: 10.1111/j.2517-6161.1995.tb02031.x
- Elkin LA, Kay M, Higgins JJ, Wobbrock JO (2021). An aligned rank transform procedure for multifactor contrast tests. *Proceedings of UIST '21*, 754ó768. DOI: 10.1145/3472749.3474784
- Kay M, Elkin L, Higgins J, Wobbrock J (2025). _ARTool: Aligned Rank Transform for Nonparametric Factorial ANOVAs_. DOI:10.5281/zenodo.594511 <https://doi.org/10.5281/zenodo.594511>, R package version 0.11.2, <https://github.com/mjskay/ARTool>.
- Kenward MG, Roger JH (1997). Small sample inference for fixed effects from restricted maximum likelihood. *Biometrics* 53: 983ó997. DOI: 10.2307/2533558.
- Kuznetsova A, Brockhoff PB, Christensen RHB (2017). lmerTest package: tests in linear mixed effects models. *Journal of Statistical Software* 82(13): 1ó26. doi:10.18637/jss.v082.i13
- Lenth R, Piaskowski J (2026). _emmeans: Estimated Marginal Means, aka Least-Squares Means_. R package version 2.0.4, <https://CRAN.R-project.org/package=emmeans>.
- Wobbrock JO, Findlater L, Gergle D, Higgins JJ (2011). The aligned rank transform for nonparametric factorial analyses using only ANOVA procedures. *Proceedings of CHI '11*, 143ó146. DOI: 10.1145/1978942.1978963.
- R Core Team (2022). R: A language and environment for statistical computing. R Foundation for Statistical Computing, Vienna, Austria. URL https://www.R-project.org/.
